# Fibronectin leucine-rich transmembrane protein 2 drives monocyte differentiation into macrophages *via* the UNC5B-Akt/mTOR axis

**DOI:** 10.3389/fimmu.2023.1162004

**Published:** 2023-04-06

**Authors:** Yaxiong Fang, Kongyang Ma, Yi-Min Huang, Yuanye Dang, Zhaoyu Liu, Yiming Xu, Xi-Long Zheng, Xiangdong Yang, Yongliang Huo, Xiaoyan Dai

**Affiliations:** ^1^ Key Laboratory of Molecular Target & Clinical Pharmacology and the State Key Laboratory of Respiratory Disease, School of Pharmaceutical Sciences, Guangzhou Medical University, Guangzhou, Guangdong, China; ^2^ Centre for Infection and Immunity Studies (CIIS), School of Medicine, Sun Yat-sen University, Shenzhen, Guangdong, China; ^3^ Medical Research Center, Guangdong Provincial Key Laboratory of Malignant Tumor Epigenetics and Gene Regulation, Sun Yat-sen Memorial Hospital, Sun Yat-sen University, Guangzhou, Guangdong, China; ^4^ School of Basic Medical Sciences, Guangzhou Medical University, Guangzhou, Guangdong, China; ^5^ Department of Biochemistry & Molecular Biology, Cumming School of Medicine, University of Calgary, Calgary, AB, Canada; ^6^ Shanghai Institute of Cardiovascular Diseases, Zhongshan Hospital, Fudan University, Shanghai, China; ^7^ Affiliated Cancer Hospital & Institute of Guangzhou Medical University, Experimental Animal Center, Guangzhou Municipal and Guangdong Provincial Key Laboratory of Protein Modification and Degradation, State Key Laboratory of Respiratory Disease, School of Basic Medical Sciences, Guangzhou Medical University, Guangzhou, Guangdong, China

**Keywords:** FLRT2, macrophage, monocyte, differentiation, UNC5B, MTOR signaling

## Abstract

Upon migrating into the tissues, hematopoietic stem cell (HSC)-derived monocytes differentiate into macrophages, playing a crucial role in determining innate immune responses towards external pathogens and internal stimuli. However, the regulatory mechanisms underlying monocyte-to-macrophage differentiation remain largely unexplored. Here we divulge a previously uncharacterized but essential role for an axon guidance molecule, fibronectin leucine-rich transmembrane protein 2 (FLRT2), in monocyte-to-macrophage maturation. FLRT2 is almost undetectable in human monocytic cell lines, human peripheral blood mononuclear cells (PBMCs), and mouse primary monocytes but significantly increases in fully differentiated macrophages. Myeloid-specific deletion of FLRT2 (*Flrt2^ΔMyel^*) contributes to decreased peritoneal monocyte-to-macrophage generation in mice *in vivo*, accompanied by impaired macrophage functions. Gain- and loss-of-function studies support the promoting effect of FLRT2 on THP-1 cell and human PBMC differentiation into macrophages. Mechanistically, FLRT2 directly interacts with Unc-5 netrin receptor B (UNC5B) *via* its extracellular domain (ECD) and activates Akt/mTOR signaling. *In vivo* administration of mTOR agonist MYH1485 reverses the impaired phenotypes observed in *Flrt2^ΔMyel^* mice. Together, these results identify FLRT2 as a novel pivotal endogenous regulator of monocyte differentiation into macrophages. Targeting the FLRT2/UNC5B-Akt/mTOR axis may provide potential therapeutic strategies directly relevant to human diseases associated with aberrant monocyte/macrophage differentiation.

## Introduction

Monocytes originate from the monocyte-dendritic cell progenitors (MDPs) in the bone marrow (BM) before entering peripheral circulation (1). Circulating monocytes can survive for several days before apoptotic cell death. Monocytes could also migrate into target tissues and then differentiate into macrophages (2). Various tissue macrophages play critical roles in immune homeostasis, inflammation, tissue repair, and diseases (3). Monocyte-to-macrophage differentiation is finely regulated at the epigenetic, transcriptional, and molecular levels (4, 5). Macrophage colony-stimulating factor (M-CSF, namely CSF-1) and granulocyte-macrophage colony-stimulating factor (GM-CSF) are the most well-known cytokines which control monocyte-to-macrophage differentiation (6, 7). Mechanistically, CSF-1 binds to its receptor CSF1R and activates phosphoinositide 3-kinase (PI3K)/protein kinase B (PKB, also known as Akt) signaling, resulting in caspase-8 and -3-mediated monocyte differentiation into macrophages (6). Extracellular receptor kinase (ERK) signaling (8) and transient nuclear factor kappa B (NF-κB) activation (9) are also involved in CSF-1-stimulated macrophage differentiation. Unlike M-CSF, GM-CSF is more specific and drives the development of lung alveolar macrophages through two transcription factors, PU.1 (10) and peroxisome proliferator-activated receptor gamma (PPARγ) (11). However, in addition to cytokines, emerging evidence shows that endogenous proteins, such as aryl hydrocarbon receptor (AHR) (12) and neutrophil serine proteases (NSPs) (13), also play critical roles in monocyte-to-macrophage differentiation through distinct mechanisms. Some intriguing questions that arise here are whether or not the additional endogenous molecules regulate monocyte/macrophage fate and what molecular mechanisms would be involved.

Fibronectin leucine-rich transmembrane protein 2 (FLRT2), first identified in a screen for extracellular matrix proteins by Lacy et al. in 1999 (14), belongs to the FLRT family, which contains other two members, FLRT1 and FLRT3. To date, most studies on FLRT2 have focused on its physiological roles in nerve, embryonic, epicardium, and vascular development *via* interactions with Unc-5 netrin receptor D (UNC5D), fibroblast growth factor receptor 2 (FGFR2), and fibronectin (15–22). Some recent studies also demonstrated the roles of FLRT2 in human tumors. For example, Bae et al. have found that FLRT2 is highly methylated in breast cancer patient tissue and inhibits the proliferation, migration, and adhesion of the breast cancer cells (23). In contrast, a recent study reveals that FLRT2 promotes cancer aggressiveness by mediating tumor-specific interendothelial adhesions (24). Other studies revealed that *Flrt2* could be used as a cancer signature gene to estimate risk stratification in early-stage stomach cancer and prostatic adenocarcinoma (PRAD) patients (25, 26). Of note, transcriptome sequencing data also showed that *Flrt2* expression was significantly higher in alveolar macrophages of tumor-bearing mice than in normal mice (27). However, the roles of FLRT2 in monocyte/macrophage differentiation remain unknown.

In the present study, we first revealed FLRT2 as a novel candidate regulator of monocyte-macrophage differentiation through comparative transcriptomic analysis. Next, we validated FLRT2 upregulation in human and mouse cell differentiation models during this process. Most importantly, we demonstrated that FLRT2 drives monocyte fate into macrophages and strengthens the ability of macrophage adhesion, migration, and phagocytosis. Furthermore, mechanistic studies showed that the binding of the FLRT2 extracellular domain (ECD) to its receptor UNC5B activates Akt/mTOR, which is essential for FLRT2-mediated monocyte-to-macrophage differentiation. Finally, we found that an mTOR agonist, MYH1485, reversed inhibited monocyte-to-macrophage differentiation, as well as impaired peritoneal macrophage functions in myeloid cell-specific FLRT2 knockout (*Flrt2^ΔMyel^*) mice. Together, we report that myeloid cell FLRT2 activates Akt/mTOR upon UNC5B interaction to drive monocyte to macrophage differentiation.

## Materials and methods

### Cell culture

Human myeloid leukemia cell lines, THP-1 and HL-60, were cultured in RPMI-1640 medium supplemented with 10% fetal bovine serum (FBS) and 1% penicillin/streptomycin (Gibco) at 37°C under an atmosphere of 5% CO_2_. A human embryonic kidney (HEK) cell line, HEK293T (ATCC), was cultured in Dulbecco′s Modified Eagle Medium (DMEM) containing 10% FBS and 1% penicillin/streptomycin at 37°C under an atmosphere of 5% CO_2_. Primary human umbilical vein endothelial cells (HUVECs, ATCC) were cultured in endothelial cell medium (ECM, 1001, ScienCell) supplemented with 5% FBS, 1% endothelial cell growth supplement, and 1% penicillin/streptomycin. Human PBMCs were purchased from LDEBIO (1501-50M, Guangzhou, China) and incubated in RPMI-1640 medium supplemented with 10% FBS, 1% penicillin/streptomycin, and 40 ng/ml M-CSF (216-MCC, R&D). Mouse PMs and BMDMs were isolated and cultured as described previously (28). For plasmid transfection, Viromer^®^ RED (VR-01LB-01, Lipocalyx) was used according to the manufacturer′s protocol.

### Mice and animal procedures

All mouse experiments in this study were approved by the Experimental Animal Center of Guangzhou Medical University (protocol no. 2019-143) and performed according to relevant ethical regulations. All mice used were bred on the C57BL/6J background. Mice were housed at 22°C in a 12 h light/dark cycle with free access to a standard chow diet and water. All mice used for experiments were from in-house mating. *Flrt2^fl/fl^* mice were custom-made by Shanghai Model Organisms and generated using C57BL/6J mice *via* a CRISPR/Cas9-based protocol. Briefly, *Cas9* mRNA and gRNA were obtained by *in vitro* transcription. A homologous recombination vector was constructed by the In-Fusion cloning method, which contained a 3.8 kb 5’-homology arm, a 3.0 kb flox region, and a 3.8 kb 3’-homology arm. *Cas9* mRNA, gRNA, and the donor vector were microinjected into fertilized zygotes of C57BL/6J mice to obtain F0 generation mice. The positive F0 generation mice were identified by PCR and mated with C57BL/6J mice to obtain positive F1 generation mice. Lysozyme 2-promoter-driven Cre (*Lyz2^Cre^*) recombinase transgenic mice were purchased from Jackson Laboratory. *Flrt2^ΔMyel^* mice were generated by crossing *Flrt2^fl/fl^* with *Lyz2^Cre^* mice. Male mice were used in all experiments between 6-8 weeks old.

### Plasmid construction and recombinant protein expression

Plasmids pLvTHM-Venus, pLvTHM-mApple, pLvTHM-shLuc, pWPI-3XFlag/Strep, and pKmyc, were kindly provided by Dr. Yongliang Huo (29). Human full-length FLRT2 was PCR amplified and subcloned into the BamHI monoclonal site of pLvTHM-Venus, pWPI-3XFlag/Strep, and pKmyc vectors. Short hairpin RNA (shRNA) sequences were selected from the MISSION shRNA library and synthesized by the TSINGKE Biological Technology company (Guangzhou, China). After initial tests, the hairpins were cloned into the ClaI-MluI sites of pLvTHM. The sequences are listed in **Table S1**. Human UNC5B cDNA (HG13606-G) was purchased from Sino Biological and cloned into the BamHI site of the pKmyc vector. Using the human FLRT2 plasmid as the template, the FLRT2 extracellular domain (hFLRT2-ECD, aa1-aa541) and FLRT2 intracellular domain (hFLRT2-ICD, aa563-aa660) were cloned into the BamHI site in the pWPI-3XFlag/Strep vector. All constructs were verified by DNA sequencing analysis (TSINGKE Biological Technology). Sequences of the primers used for plasmid construction are listed in **Table S2**.

### *In vitro* and *in vivo* monocyte-to-macrophage differentiation models

THP-1 and HL-60 cells were treated *in vitro* with 100 ng/ml phorbol-12-myristate-13-acetate (PMA, P8139, Sigma) for 24 h. Primary BM cells were isolated from C57BL/6J mice and treated with 25 ng/ml M-CSF for 5 days. For *in vivo* monocyte-to-macrophage differentiation, 1 ml of 4% sterile thioglycollate broth (211716, BD Biosciences) was intraperitoneally (i.p.) injected into mice, and PMs were collected at the end of day 3. To be differentiated into macrophages, human PBMCs were induced by 40 ng/ml M-CSF for 7 d.

### Morphological analysis

Morphological changes in cells were observed using a phase-contrast light microscope (Leica, DMI 4000B).

### Cell adhesion assay

Red fluorescent protein (RFP)-expressing HUVECs were seeded onto slides in 60 mm dishes and grown to 100% confluence. After that, HUVECs were washed with PBS and cocultured with 5×10^6^ PBMCs or THP-1 cells expressing green fluorescent protein (GFP), GFP-hFLRT2, negative control GFP-shRNA (GFP-shNC), or GFP-shFLRT2 #3 or 5×10^6^ calcein AM-labeled PMs for 6 h. After washing, THP-1 cells, PBMCs, or PMs adhering to the HUVEC monolayer were examined with a stereoscopic fluorescence microscope (Leica, M205FA). Relative GFP fluorescence intensities (AU) were quantified using Image J. The control value was defined as 1-fold.

### *In vitro* cell migration assay

First, 1×10^5^ PBMCs or THP-1 cells were plated in the upper chamber of 24-well Transwell plates (3422, Corning). RPMI-1640 medium containing 10% FBS was added to the lower chambers. Cells were maintained in culture conditions at 37°C and allowed to migrate for 48 h. Next, unmigrated cells in the upper chamber were wiped off with a cotton tip. Migrated cells on the underside of the filter were fixed and stained with 0.1% crystal violet. Images were then captured with an inverted fluorescence microscope (Zeiss, Axio Observer5). The number of migrated cells was counted in nine randomly chosen fields per insert.

### Phagocytosis assay

1×10^6^ PBMCs or THP-1 cells were cultured in the presence or absence of 100 ng/ml PMA and incubated with 30 μg/ml Texas red-conjugated zymosan particles (Z23374, Invitrogen) for 2 h or 12 h. Cells were viewed for internalization of the particles by fluorescence microscopy. For *in vivo* phagocytosis, 1 ml of 4% sterile thioglycollate broth was injected i.p. into mice. After 3 days, 600 μg (dissolved in 100 μl PBS) of zymosan particles were injected i.p. After 2 h, PMs were isolated. The phagocytic particles of macrophages were observed with an inverted microscope (Leica, DMI 4000 B). The percentages of cells containing zymosan among total cells were calculated.

### RNA extraction and quantitative RT-PCR

Total RNA was isolated using RNAex (AG21101, Accurate Biology), and RNA concentration was measured on the NanoDrop one Spectrophotometer according to the manufacturer′s instructions (Thermo). cDNA was generated using Evo M-MLV RT Premix reagent (AG11706, Accurate Biology). The primers for qPCR used in this study are listed in **Table S3**. Gene expression was normalized using glyceraldehyde 3-phosphate dehydrogenase (*Gapdh*), and relative expression of genes was calculated using the 2^–ΔΔCt^ method.

### Western blotting and immunoprecipitation

Cell lysates were subjected to immunoblot analysis, as described previously (30). The protein content was processed by the BCA protein assay (P0009, Beyotime). 50 μg total protein was loaded onto SDS-PAGE and then transferred to a nitrocellulose (NC) membrane (66485, PALL). Membranes were blocked with 5% skimmed milk for 1 h at room temperature and then incubated with primary antibodies and HRP-conjugated secondary antibodies. Protein bands were visualized by enhanced chemiluminescence (ECL, 32016, Thermo-Fisher), and the densitometry of the blots was detected using an Amersham™ Imager 600 (GE Healthcare, Chicago, IL, USA). GAPDH or β-actin served as an internal control. Bands were quantitatively analyzed with Image J. The background noise was subtracted from the calculation.

For IP experiments, 5×10^6^ cells were subjected to 1 ml lysis buffer (25 mM HEPES pH 7.4, 150 mM NaCl, 0.5 mM EDTA, 5 mM MgCl_2_, 0.5% Triton X-100, 2 mM DTT, 1 mM PMSF, 1 mg/ml aprotinin, 1 mg/ml leupeptin, 1 mg/ml pepstatin) on ice for 10 min. To obtain the supernatant, cell lysates were centrifuged for 20 min at 12,000 rpm at 4°C in a microcentrifuge. Anti-FLAG^®^ M2 Magnetic Beads were blocked in 5% BSA solution for 1 h, then added to the supernatant, and incubated for 12 h at 4°C. After washing three times with ice-cold high-salt buffer (25 mM HEPES pH 7.4, 450 mM NaCl, 0.5 mM EDTA, 5 mM MgCl_2_, 0.5% Triton X-100, 1 mM DTT, 1 mM PMSF), samples were placed on a magnetic stand to remove supernatant, and then washed twice with wash buffer (25 mM HEPES pH 7.4, 150 mM NaCl, 0.5 mM EDTA, 5 mM MgCl_2_, 1 mM DTT). 2 × loading buffer was added, and the magnetic beads were boiled for 10 min. Appropriate primary and secondary antibodies were selected for subsequent immunoblot analysis. The antibodies used for immunoblot and IP analyses are listed in **Table S1**.

### Immunofluorescence

Cells were plated on glass coverslips, fixed with 4% paraformaldehyde, permeabilized with 0.3% Triton X-100, and blocked with 10% Western Blocking Reagent (11921673001, Roche) for 1 h at room temperature. Primary antibodies were incubated with the cells overnight at 4°C. Slides were rinsed with washing buffer and incubated with Alexa 488/555/647 conjugated secondary antibodies for 1 h at 1:1000 dilution. The antibodies used for IF are listed in **Table S1**. Nuclei were stained with 1:500 DAPI (D1306, Invitrogen). Slides were mounted and visualized using a confocal laser scanning microscope (Leica, TCS SP8).

### RNA-sequencing analysis

THP-1 cells were treated with dimethylsulfoxide (DMSO) or 100 ng/ml PMA for 24 h. Briefly, RNA was extracted using the RNeasy midi-kit, enriched by magnetic beads with Oligo (dT), and RNA quality was assessed using an Agilent 2100 Bioanalyzer. Library preparation was performed using the TruSeq Stranded mRNA kit (Illumina), and single-end 125 bp reads were generated on an Illumina HiSeqTM 2500. RNA-seq analysis was conducted by Shanghai OE Biotech. Co., Ltd. China.

### Protein preparation and mass spectrometry

HEK293T cells were transfected with control vector or Flag-UNC5B for 48 h. Protein samples were collected using anti-Flag antibody and used to conduct liquid chromatography-tandem MS (LC-MS/MS) experiments using the Easy nLC 1200 system (ThermoFisher). DDA (data-dependent acquisition) mass spectrum techniques were used to acquire tandem MS data on a ThermoFisher Q Exactive mass spectrometer (ThermoFisher, USA) fitted with a Nano Flex ion source. The LC-MS/MS data were analyzed for protein identification and quantification using PEAKS Studio 8.5. The local false discovery rate at PSM was 1.0% after searching against the *Homo sapiens* database with a maximum of two missed cleavages. The following settings were selected: Oxidation (M), Acetylation (Protein N-term), Deamidation (NQ), Pyro-glu from E, Pyro-glu from Q for variable modifications as well as fixed Carbamidomethylation of cysteine. Precursor and fragment mass tolerance were set to 10 ppm and 0.05 Da, respectively. The LC-MS/MS data were uploaded to ProteomeXchange. The shareable link is: http://proteomecentral.proteomexchange.org/cgi/GetDataset?ID=PXD040319. PXD number: PXD040319.

### Sample preparation for flow cytometry

Blood was collected through cardiac puncture from terminally anesthetized mice. Peritoneal lavage was obtained by injecting 8 ml PBS containing 2 mM EDTA into the peritoneal cavity, and the washout was collected. Mice were then perfused with PBS *via* the left ventricle. BM was prepared by flushing the BM from the femur and tibia of mice, and then the cell suspension was passed through a 70 μm cell strainer. Red blood cells were lysed.

### Flow cytometric analysis

For multicolor flow cytometric analysis, single-cell suspensions from the peritoneal cavity, peripheral blood, and BM were prepared. The Zombie Aqua fixable viability dye (423102, BioLegend) was used to exclude dead cells, and rat monoclonal anti-CD16/32 antibody (an Fc blocker) was incubated for 30 min at room temperature to block nonspecific antibody binding. Then, single-cell suspensions were labeled with fluorescent-dye conjugated antibodies: Pacific blue-conjugated anti-mouse CD45, FITC-conjugated anti-mouse CD11b, and APC-conjugated anti-mouse F4/80. APC/Cy7-conjugated anti-mouse CD19 antibodies were added to exclude B cells in peritoneal cell suspensions. The antibodies used for flow cytometry are listed in **Table S1**. All flow cytometric data were acquired using a BD LSR Fortessa cytometer (BD Biosciences, CA, USA) and analyzed with the FlowJo software (BD Biosciences).

### Statistics

Statistical analysis for non-sequencing data was performed using Prism 8 software (GraphPad). Data are presented as means ± standard deviation (SD). Unpaired, 2-tailed Student *t*-test was used for comparing 2 groups. ND, not detected. NS, not significant.

Statistical significance is indicated as follows: ^*^ *p* < 0.05, ^**^ *p* < 0.01, ^***^ *p* < 0.001, and ^****^ *p* < 0.0001.

## Results

### FLRT2 expression is dramatically upregulated as human and mouse monocytes differentiate into macrophages *in vitro* and *in vivo*

**Figure 1:**
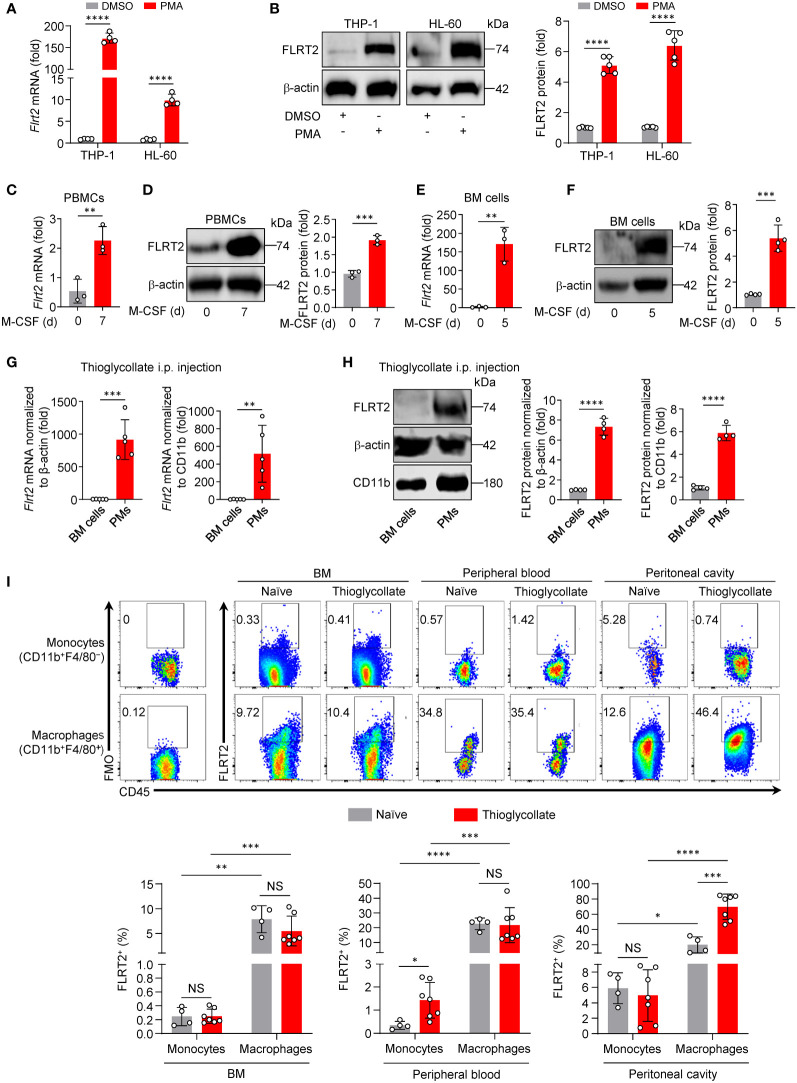
FLRT2 is significantly induced during monocyte-to-macrophage differentiation *in vitro* and *in vivo*. **(A, B)** THP-1 or HL-60 cells were treated with DMSO or PMA (100 ng/ml) for 24 h. FLRT2 mRNA and protein levels were detected by qPCR (**A**, n = 4) and immunoblot (**B**, n = 5) analyses, respectively. **(C, D)** qPCR analysis of *Flrt2* mRNA (**C**, n = 3) and immunoblot analysis of FLRT2 protein (**D**, n = 3) in human PBMCs induced by M-CSF (40 ng/ml) for 0 or 7 days. **(E, F)** qPCR analysis of *Flrt2* mRNA (**E**, n = 3) and immunoblot analysis of FLRT2 protein (**F**, n = 4) in bone marrow (BM) cells induced by M-CSF (25 ng/ml) for 0 or 5 days. **(G, H)** qPCR analysis of *Flrt2* mRNA (**G**, n = 5 mice per group) and immunoblot analysis of FLRT2 protein (**H**, n = 4 mice per group) in BM cells and peritoneal macrophages (PMs) isolated from C57BL/6J mice after intraperitoneal (i.p.) thioglycollate injection. mRNA and protein levels were normalized to both β-actin and CD11b. **(I)** Representative flow cytometric profiles and data plots showing the frequencies of FLRT2^+^CD45^+^CD11b^+^F4/80^−^ monocytes and FLRT2^+^CD45^+^CD11b^+^F4/80^+^ macrophages in the BM, peripheral blood, and peritoneal cavity of C57BL/6J mice injected i.p. with or without thioglycollate for 3 days (n = 4 or 7 mice per group). Data are means ± SD. *P* values were determined using unpaired, two-tailed Student’s *t*-tests. NS, not significant. ^*^ *P* < 0.05, ^**^ *P* < 0.01, ^***^ *P* < 0.001, ^****^ *P* < 0.0001.

Phorbol-12-myristate-13-acetate (PMA) induces THP-1 (a human monocyte cell line) cell differentiation, representing a valuable model for studying monocyte-to-macrophage differentiation *in vitro* (31). To identify new endogenous candidate molecules that regulate this process, we performed unbiased RNA-seq analysis in differentiated and undifferentiated THP-1 cells. As shown in **Supplementary Figure 1A**, genes that are differentially expressed (DEGs) include 2,938 upregulated genes (>2-fold) and 2,717 downregulated genes (<2-fold). Surprisingly, the FLRT family (FLRT1, 2, and 3), involved in the development of vertebrate embryos (18, 19, 32–34), shows distinct patterns of expression in this monocyte-to-macrophage differentiation system (**Supplementary Figure 1B**). As shown in **Supplementary Figure 1B**, we observed a 474-fold increase in *Flrt2* mRNA expression and a 3.5-fold decrease in *Flrt1* mRNA expression. However, *Flrt3* expression was undetectable in both undifferentiated and differentiated THP-1 cells (**Supplementary Figure 1B**). Since the changes in *Flrt2* mRNA levels were much more significant than those of *Flrt1*, we decided to focus on FLRT2. Using qPCR and immunoblot analyses, we confirmed a dramatical FLRT2 increase in PMA-differentiated THP-1 cells, as well as HL-60 (a human myeloid leukemia cell line) cells (**Figures 1A, B**). We also observed higher expression of FLRT2 in M-CSF-treated human PBMCs (**Figures 1C, D**).

To determine whether the upregulation of FLRT2 was also present during mouse monocyte-to-macrophage differentiation, we isolated primary BM cells from C57BL/6J mice. The cells were then co-cultured with M-CSF, a well-known macrophage differentiation inducer (35). As shown in **Figures 1E, F**, M-CSF significantly induced FLRT2 mRNA and protein expression in BM cells. We subsequently examined FLRT2 expression *in vivo* in mice during the differentiation. As shown in **Figures 1G, H**, we observed that FLRT2 mRNA and protein levels were elevated in peritoneal macrophages (PMs) collected from mice intraperitoneally (i.p.) injected with thioglycollate for 3 days compared to BM cells from the same mice.

To gain insight into the potential roles of FLRT2 during monocyte/macrophage development *in vivo*, we analyzed organ distribution and major cell populations of FLRT2-expressing subsets of C57BL/6J mice injected i.p. with thioglycollate for 3 days. In **Figure 1I**, flow cytometry data clearly showed a significantly increased frequency of FLRT2^+^ cells in CD45^+^CD11b^+^F4/80^+^ macrophages of BM, peripheral blood, and peritoneal cavity when compared to that in CD45^+^CD11b^+^F4/80^−^ monocytes from these tissues. Notably, thioglycollate injection markedly elevated the frequency of FLRT2^+^CD45^+^CD11b^+^F4/80^+^ macrophages (69.73% ± 16.70%) versus FLRT2^+^CD45^+^CD11b^+^F4/80^−^ monocytes (19.86% ± 10.46%) in the peritoneal cavity, implying that FLRT2 holds an essential role in peritoneal macrophage generation (**Figure 1I**). However, the comparable frequency of FLRT2^+^CD45^+^CD11b^+^F4/80^+^ macrophages was shown in BM and peripheral blood of mice treated with or without thioglycollate (**Figure 1I**). Together, FLRT2^+^ cells are mostly CD45^+^CD11b^+^F4/80^+^ macrophages, and thioglycollate administration induces FLRT2 expression in peritoneal macrophages. Taken together, we demonstrated that FLRT2 expression is significantly induced during human and mouse monocyte-to-macrophage differentiation *in vitro* and *in vivo*.

### Myeloid cell-specific deletion of FLRT2 inhibits peritoneal macrophage development and functions in mice *in vivo*

To explore the role of FLRT2 in monocyte/macrophage development, we generated *Flrt2^ΔMyel^* mice by crossbreeding *Flrt2^fl/fl^* mice with *Lyz2^Cre^* mice. In *Flrt2^fl/fl^; Lyz2^Cre+^* mice, *Lyz2^Cre^* recombinase deleted the floxed *Flrt2* gene in myeloid cells. The *Flrt2^fl/fl^* littermates served as the control group (**Supplementary Figure 2A**). All mice were genotyped with PCR (**Supplementary Figure 2B**). To confirm the efficiency of FLRT2 deletion in myeloid cells, we isolated bone marrow-derived macrophages (BMDMs) and PMs from *Flrt2^fl/fl^* and *Flrt2^ΔMyel^* mice. As shown in **Supplementary Figure 2C**, *Flrt2* mRNA was undetectable in BMDMs and PMs from *Flrt2^ΔMyel^* mice when compared with *Flrt2^fl/fl^* mice. FLRT2 deficiency was further confirmed by immunoblots (**Supplementary Figure 2D**). In contrast to macrophages, no significant difference in FLRT2 protein levels was observed in the liver, kidney, heart, and lung from *Flrt2^fl/fl^* and *Flrt2^ΔMyel^* mice (**Supplementary Figure 2E**). In sum, FLRT2 was specifically and efficiently deleted in myeloid cells.

**Figure 2:**
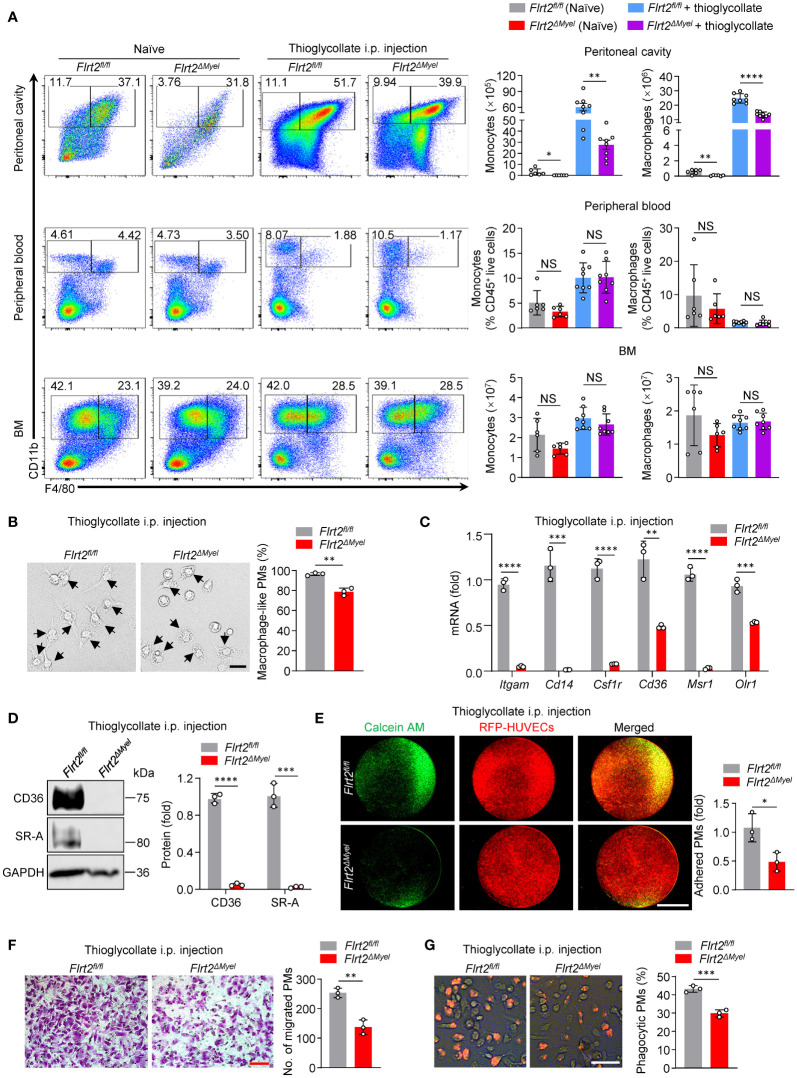
Myeloid cell-specific FLRT2 knockout (*Flrt2^ΔMyel^*) leads to decreased peritoneal monocyte-to-macrophage differentiation and impaired peritoneal macrophage functions *in vivo*. **(A)** Representative flow cytometric profiles and data plots showing the absolute numbers of CD45^+^CD11b^+^F4/80^−^ monocytes and CD45^+^CD11b^+^F4/80^+^ macrophages in the peritoneal cavity and BM, and the frequencies of CD45^+^CD11b^+^F4/80^−^ monocytes and CD45^+^CD11b^+^F4/80^+^ macrophages in peripheral blood of *Flrt2^fl/fl^* and *Flrt2^ΔMyel^* mice treated with or without i.p. injection of thioglycollate for 3 days (n = 6 or 8 mice per group). **(B)** Phase-contrast microscopy images showing morphological alterations of PMs isolated from *Flrt2^fl/fl^* and *Flrt2^ΔMyel^* mice (n = 3 mice per group). Scale bar, 50 μm. **(C)** qPCR analysis of *Itgam*, *Cd14*, *Csf1r*, *Cd36*, *Msr1*, and *Olr1* mRNA in PMs isolated from *Flrt2^fl/fl^* and *Flrt2^ΔMyel^* mice (n = 3 mice per group). **(D)** CD36 and SR-A protein levels in PMs isolated from *Flrt2^fl/fl^* and *Flrt2^ΔMyel^* mice were determined by immunoblot analysis (n = 3 mice per group). **(E)** PMs were collected from *Flrt2^fl/fl^* and *Flrt2^ΔMyel^* mice and then co-cultured with HUVECs expressing RFP for 6 h, followed by fluorescent microscopy (n = 3 mice per group). Scale bar, 5 mm. **(F)** PMs were isolated from *Flrt2^fl/fl^* and *Flrt2^ΔMyel^* mice and seeded in a Boyden chamber for 48 h, followed by crystal violet staining (n = 3 mice per group). Scale bar, 100 μm. **(G)** Zymosan was injected i.p. into *Flrt2^fl/fl^* and *Flrt2^ΔMyel^* mice for 2 h, and PMs were collected and placed in a 6-well plate for 6 h. The phagocytic particles were observed under a laser confocal scanning microscope (n = 3 mice per group). Scale bar, 50 μm. Data are means ± SD. *P* values were determined using unpaired, two-tailed Student’s *t*-tests. NS, not significant. ^*^ *P* < 0.05, ^**^ *P* < 0.01, ^***^ *P* < 0.001, ^****^ *P* < 0.0001.

In order to understand which biological processes, cellular components, and molecular functions in macrophages were greatly affected by the absence of FLRT2, we isolated PMs from *Flrt2^fl/fl^* and *Flrt2^ΔMyel^* mice, and determined the transcriptome using RNA-seq. A total of 4,308 genes differentially expressed in PMs were identified, including 2,131 upregulated genes and 2,177 downregulated genes (**Supplementary Figure 3A**). Gene Ontology (GO) term analysis for genes in PMs revealed that those downregulated DEGs were predominantly enriched in biological processes rather than cellular components and molecular functions (**Supplementary Figure 3B**). One of the significantly affected biological pathways of note is the developmental process (**Supplementary Figure 3B**). To directly assess the role of FLRT2 in the development of monocytes into macrophages *in vivo*, we established a monocyte-macrophage differentiation model by i.p. injecting thioglycollate into *Flrt2^fl/fl^* and *Flrt2^ΔMyel^* mice. In **Figure 2A**, the data showed that myeloid cell-specific FLRT2 deletion led to a reduced cell number of CD11b^+^F4/80^+^ macrophages in the peritoneal cavity but not in peripheral blood and BM under both naïve and thioglycollate-stimulated conditions. Surprisingly, we also observed a significant decrease in the cell number of CD11b^+^F4/80^−^ monocytes in the peritoneal cavity of in *Flrt2^ΔMyel^* mice compared with *Flrt2^fl/fl^* mice under both naïve and thioglycollate-stimulated conditions, suggesting FLRT2 may regulate cell survival (**Figure 2A**). Since monocytes are loosely attached cells with a round shape, and mature macrophages are firmly anchored cells with a spread and irregular shape (36), we were able to observe morphological changes in PMs using a phase-contrast light microscope. Phase-contrast images showed that myeloid cell-specific FLRT2 deletion reduced the percentage of mature PMs (**Figure 2B**). Moreover, qPCR analysis revealed that FLRT2-null PMs showed a significant decrease in macrophage marker genes, including *Itgam*, *Cd14*, *Csf1r*, *Cd36*, *Msr1*, and *Olr1* (**Figure 2C**). Immunoblot analysis demonstrated that FLRT2-deficient PMs exhibited reduced CD36 and SR-A protein levels (**Figure 2D**). Together, our murine studies suggest that FLRT2 deficiency limits peritoneal monocyte-to-macrophage differentiation *in vivo*.

It is known that macrophages differentiated from monocytes gain several unique functions, such as adhesion, migration, and phagocytosis (37). Our RNA-seq data showed that many genes involved in macrophage adhesion and phagocytosis were aberrantly regulated in FLRT2*-*deficient PMs, indicating the functional defects of macrophages caused by FLRT2 deletion (**Supplementary Figures 3C, D**). To confirm it, we analyzed the functions of PMs isolated from *Flrt2^fl/fl^* and *Flrt2^ΔMyel^* mice. First, we assessed direct PM adhesion towards to HUVECs. As shown in **Figure 2E**, macrophage-specific FLRT2 loss led to a significant decrease in the adhesive ability of PMs to HUVECs. Next, we assessed cell migration potential by employing Boyden chamber analysis. The results showed reduced migration capability of FLRT2-deficient PMs (**Figure 2F**). Finally, we examined phagocytic capacity using a zymosan uptake assay, a widely used model for macrophage microbe recognition (38). The *in vivo* zymosan uptake assay showed attenuated phagocytosis capability in PMs from *Flrt2^ΔMyel^* mice relative to *Flrt2^fl/fl^* controls (**Figure 2G**). Collectively, myeloid cell-specific FLRT2 deficiency moderated peritoneal macrophage functions.

### FLRT2 drives the differentiation of human monocytes into macrophages and strengthens the differentiated macrophage functions

To understand the effects of FLRT2 on monocyte-to-macrophage differentiation in a human monocytic cell line, we transfected THP-1 cells with either full-length human FLRT2 (hFLRT2) or control vector. Increased macrophage-like cell frequency was observed in FLRT2-overexpressed THP-1 cells (**Supplementary Figure 4A**). More importantly, FLRT2 overexpression correlated with increased macrophage signature genes (**Supplementary Figure 4B**). Immunoblot analysis confirmed that FLRT2 promoted CD36 and SR-A protein levels (**Supplementary Figure 4C**). The FLRT2 overexpression was verified in **Supplementary Figures 4B, C**. Next, we performed loss-of-function experiments using shFLRT2 #3, as validated in **Supplementary Figures 5**. As expected, FLRT2 silencing correlated with a decreased percentage of mature macrophages in PMA-induced THP-1 cells (**Supplementary Figure 4D**). FLRT2 knockdown significantly decreased macrophage marker gene expression and protein levels in the cells (**Supplementary Figures 4E, F**). The knockdown of FLRT2 in THP-1 cells was also confirmed (**Supplementary Figures 4E, F**). Functional assays supported a promoting role of FLRT2 in the adhesive, migratory, and phagocytotic capability of THP-1 cells (**Supplementary Figures 4G–L**).

**Figure 3:**
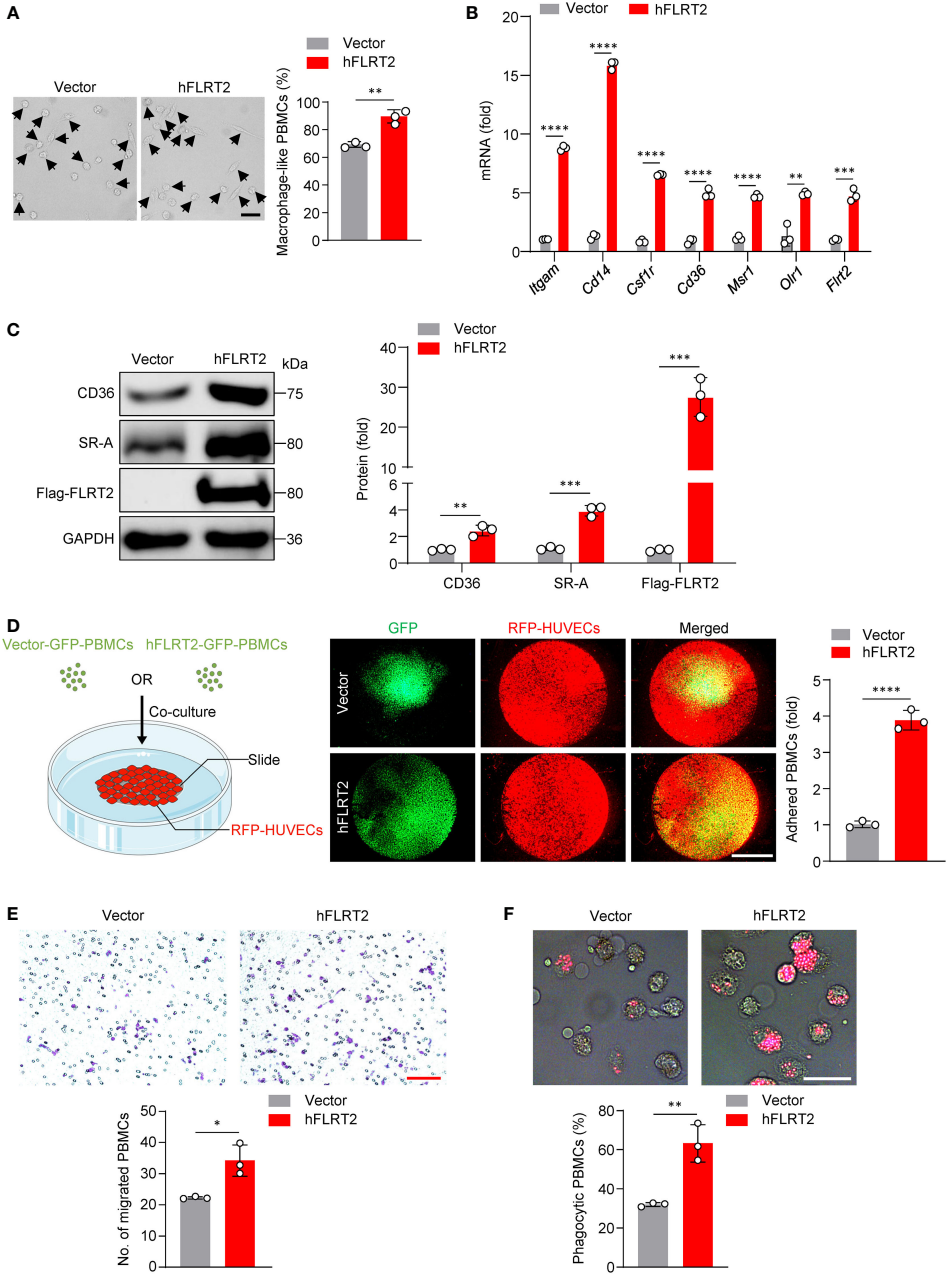
FLRT2 induces PBMC differentiation into macrophages and enhances the adhesion, migration, and phagocytosis of M-CSF-primed PBMC-derived macrophages. **(A–C, E, F)** Human PBMCs were transfected with control or hFLRT2 vector for 48 h. **(A)** Representative phase-contrast light microscopy images showing morphological alterations of PBMCs transfected as indicated (n = 3). Scale bar, 50 μm. **(B)** qPCR analysis of *Itgam*, *Cd14*, *Csf1r*, *Cd36*, *Msr1*, *Olr1*, and *Flrt2* mRNA in PBMCs cells transfected as indicated (n = 3). **(C)** Immunoblot analysis of CD36, SR-A, and Flag-FLRT2 proteins in PBMCs transfected as indicated (n = 3). **(D)** HUVECs expressing RFP were co-cultured with PBMCs expressing vector-GFP or hFLRT2-GFP. After 6 h co-culture, pictures were taken using a fluorescence microscope (n = 3). Scale bar, 5 mm. **(E)** Transwell cell migration assays were performed, and numbers of migrated cells were counted (n = 3). Scale bar, 100 μm. **(F)** Phagocytosis assays were performed by culturing the cells in Texas red-conjugated zymosan particles for 2 h at 37°C (n = 3). Cells were viewed for internalization of the particles by fluorescence microscopy. Scale bar, 50 μm. Data are means ± SD. *P* values were determined using unpaired, two-tailed Student’s *t*-tests. ^*^ *P* < 0.05, ^**^ *P* < 0.01, ^***^ *P* < 0.001, ^****^ *P* < 0.0001.

To study the role of FLRT2 on primary human monocyte-to-macrophage differentiation *in vitro*, we transfected PBMCs with either hFLRT2 or control vector. The results showed an increase in macrophage-like cell frequency in FLRT2-overexpressed PBMCs relative to control cells (**Figure 3A**). In addition, qPCR data indicated that FLRT2 overexpression was accompanied by a significant increase in macrophage signature genes, including *Itgam*, *Cd14*, *Csf1r*, *Cd36*, *Msr1*, as well as *Olr1* (**Figure 3B**). Consistently, immunoblot analysis demonstrated that FLRT2 elevated CD36 and SR-A protein levels (**Figure 3C**). The overexpression of FLRT2 in PBMCs was verified in **Figures 3B, C**. In line with our mouse findings, FLRT2 overexpression promoted M-CSF-primed PBMC-derived macrophage adhesion to HUVECs (**Figure 3D**). Boyden chamber analysis showed that FLRT2 overexpression increased the migratory ability of M-CSF-primed PBMC-derived macrophages (**Figure 3E**). Furthermore, FLRT2 overexpression increased the phagocytosis capability of M-CSF-primed PBMC-derived macrophages (**Figure 3F**). Collectively, these results demonstrate that FLRT2 positively regulates the generation of human THP-1 cell and PBMC-derived macrophages and enhances the capability of adhesion, migration, and phagocytosis of THP-1 cell- and PBMC-derived macrophages.

### FLRT2-induced THP-1 differentiating into macrophages is dependent on UNC5B

**Figure 4:**
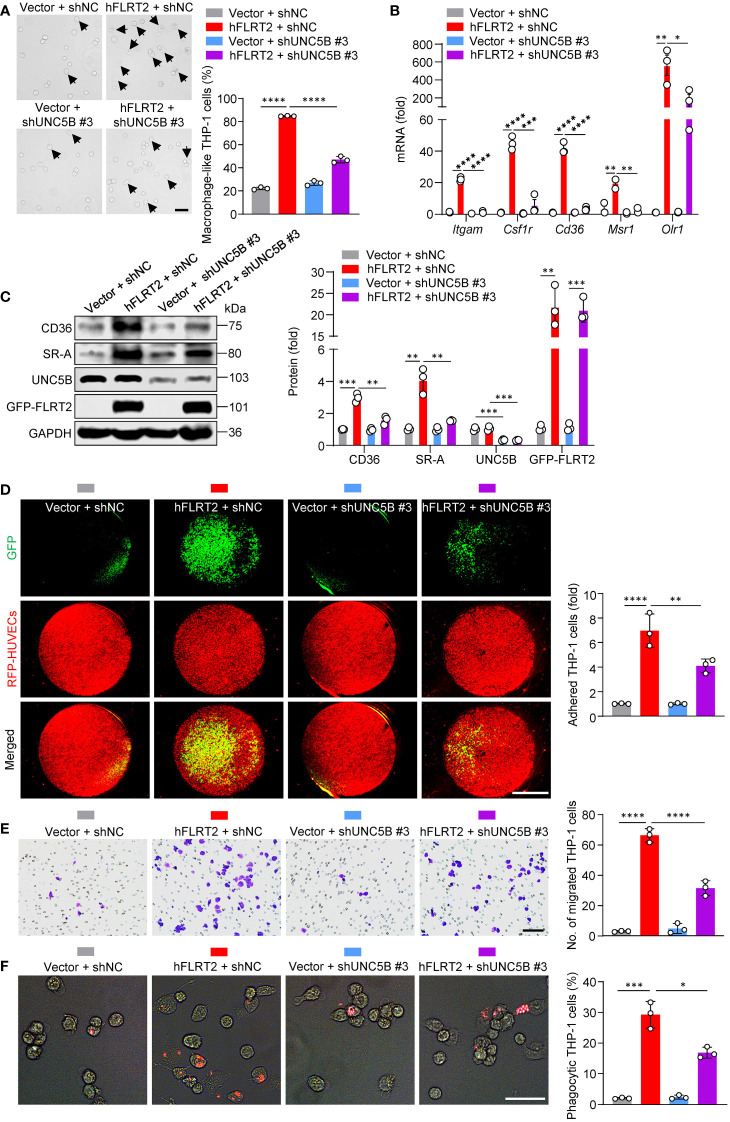
UNC5B mediates FLRT2-promoted THP-1 cell maturation into macrophages. FLRT2 was overexpressed by transfecting the hFLRT2 vector, and UNC5B was silenced by transfecting UNC5B-specific shRNA into THP-1 cells. **(A)** Representative phase-contrast light microscopy images showing morphological alterations of THP-1 cells transfected as indicated (n = 3). Scale bar, 50 μm. **(B)** qPCR analysis of *Itgam*, *Csf1r*, *Cd36*, *Msr1*, and *Olr1* mRNA in THP-1 cells transfected as indicated (n = 3). **(C)** Immunoblot analysis of CD36, SR-A, UNC5B, and GFP-FLRT2 proteins in THP-1 cells transfected as indicated (n = 3). **(D)** HUVECs expressing RFP were co-cultured with THP-1 cells transfected as indicated. After 6 h co-culture, pictures were taken using a fluorescence microscope (n = 3). Scale bar, 5 mm. **(E)** Transwell cell migration assay was performed in THP-1 cells transfected as indicated, and the numbers of the migrated cells were counted (n = 3). Scale bar, 100 μm. **(F)** Phagocytosis assays were performed by culturing the cells transfected as indicated in Texas red-conjugated zymosan particles for 2 h at 37°C (n = 3). Cells were observed for internalization of the particles by fluorescence microscopy. Scale bar, 50 μm. Data are means ± SD. *P* values were determined using unpaired, two-tailed Student’s *t*-tests. ^*^ *P* < 0.05, ^**^ *P* < 0.01, ^***^ *P* < 0.001, ^****^ *P* < 0.0001.

We processed to dissect how FLRT2 promotes the differentiation of monocytes into macrophages. As a single transmembrane protein, FLRT2 functions through several receptors, including FGFR2 (39), UNC5C (40), UNC5D (21), and UNC5B (16). Our RNA-seq results showed that *Fgfr2* and *Unc5d* gene expressions were undetectable in both DMSO- and PMA-treated THP-1 cells; *Unc5c* was very low in DMSO-treated undifferentiated THP-1 cells and almost undetectable in PMA-treated differentiated THP-1 cells (**Supplementary Figure 6A**). In addition, PMA did not significantly regulate *Unc5c* gene levels (**Supplementary Figure 6A**). However, *Unc5b* was upregulated more than 10-fold by PMA (**Supplementary Figure 6A**). Strikingly, the substantial induction of *Unc5b* expression was further verified in various human and mouse monocyte-to-macrophage differentiation models (**Supplementary Figure 6B–G**). Thus, we speculated that FLRT2-promoted monocyte-to-macrophage differentiation depends on UNC5B. To test it, we selected shUNC5B #3 to knockdown UNC5B expression (**Supplementary Figure 7**). As shown in **Figure 4A**, FLRT2-overexpressed THP-1 cells showed an increased frequency of macrophage-like cells while knockdown of UNC5B reversed this effect. In addition, UNC5B ablation attenuated FLRT2-driven expression of macrophage marker genes, including *Itgam*, *Csf1r*, *Cd36*, *Msr1*, and *Olr1* (**Figure 4B**). Consistently, the lack of UNC5B downregulated FLRT2-increased CD36 and SR-A protein levels (**Figure 4C**). Significantly, UNC5B knockdown markedly reversed FLRT2 overexpression-enhanced THP-1 cell functions, including adhesion, migration, and phagocytosis (**Figures 4D–F**). Together, our data suggested that UNC5B is essential for FLRT2-induced THP-1 cell differentiation into macrophages and the functions of these cells.

### The binding of the FLRT2 extracellular domain to UNC5B is required for FLRT2-mediated THP-1 cell maturation into macrophages

**Figure 5:**
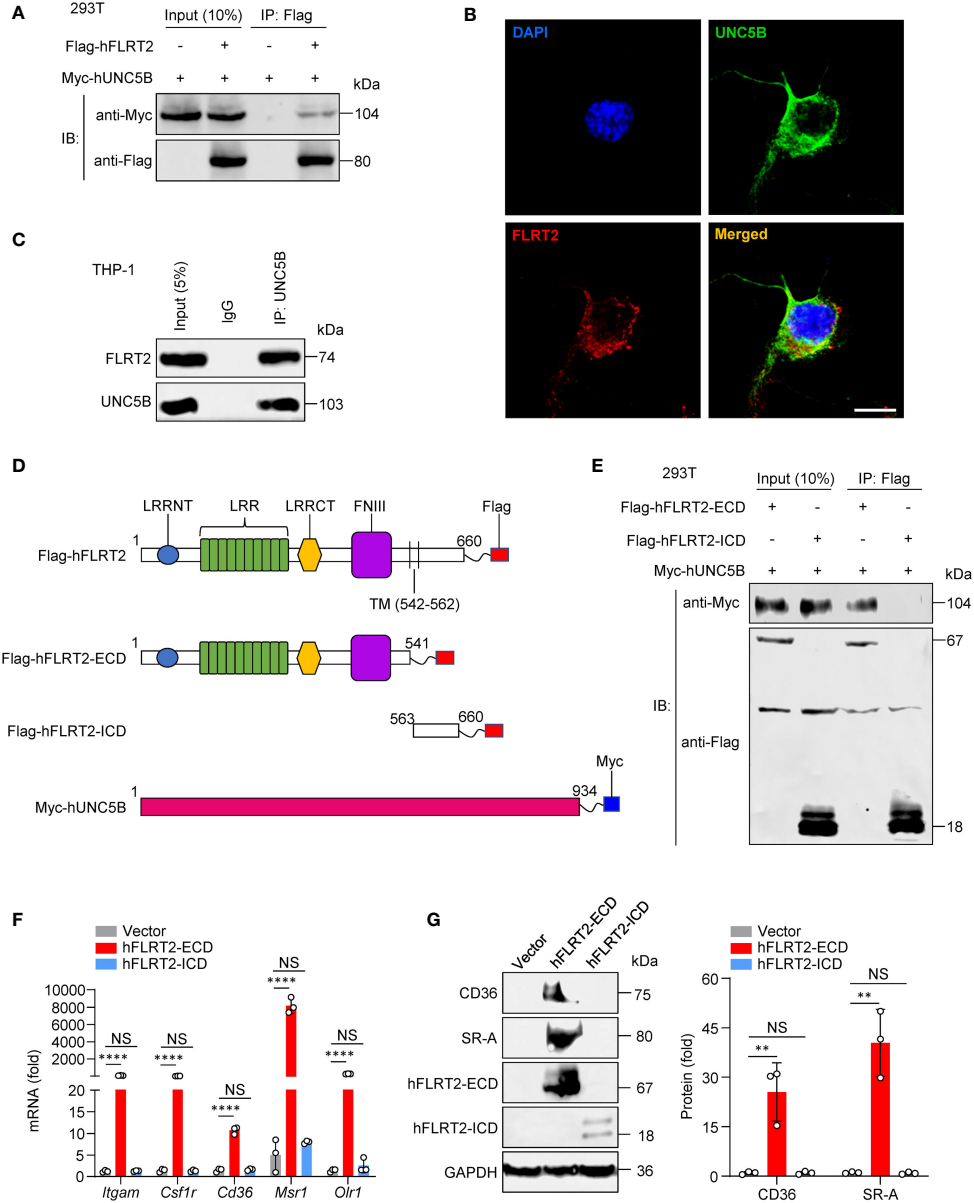
The direct interaction of the FLRT2 ECD with UNC5B is required for FLRT2-promoted THP-1 cell differentiation into macrophages. **(A)** Myc-tagged hUNC5B and/or Flag-tagged hFLRT2 were transfected into HEK293T cells. Immunoprecipitation samples were collected using anti-Flag antibody, followed by immunoblotting for Myc and Flag (n = 3). **(B)** Immunolabeling of FLRT2 and UNC5B in PMA-treated THP-1 cells showing the colocalization of endogenous UNC5B and FLRT2. Scale bar, 10 μm. **(C)** UNC5B was immunoprecipitated from PMA-incubated THP-1 cells, followed by immunoblotting for FLRT2 and UNC5B (n = 3). **(D)** Schematic illustration of FLRT2 and UNC5B constructs. LRRNT, leucine-rich repeat N-terminal domain. LRR, leucine-rich repeat. LRRCT, leucine-rich repeat C-terminal domain. FNIII, fibronectin type III domain. **(E)** UNC5B interacts with hFLRT2-ECD but not hFLRT2-ICD (n = 3). **(F, G)** THP-1 cells were transfected with control, human FLRT2 extracellular domain (hFLRT2-ECD) or human FLRT2 intracellular domain (hFLRT2-ICD) vector for 48 h, followed by qPCR **(F)** and immunoblot **(G)** analyses. Data are means ± SD. *P* values were determined using unpaired, two-tailed Student’s *t*-tests. NS, not significant. ^**^ *P* < 0.01, ^****^ *P* < 0.0001.

Based on the results that the blockade of UNC5B abolished the FLRT2-triggered THP-1 differentiating into macrophages, we sought to determine whether FLRT2 interacted with UNC5B to control this process. Through co-immunoprecipitation (co-IP) experiments, we proved the interaction between exogenously expressed hFLRT2 and hUNC5B (**Figure 5A**). We further found that FLRT2 co-localized with UNC5B in the cell membrane of PMA-treated THP-1 cells (**Figure 5B**). Notably, we confirmed the direct interaction of endogenous FLRT2 and UNC5B in THP-1 cells (**Figure 5C**). To further identify which domain of FLRT2 is essential for the binding, we constructed Flag-tagged full-length human FLRT2 (hFLRT2), FLRT2 extracellular domain (hFLRT2-ECD), FLRT2 intracellular domain (hFLRT2-ICD), and Myc-tagged full-length human UNC5B (hUNC5B) vectors (**Figure 5D**). Co-IP results revealed that UNC5B co-immunoprecipitated with hFLRT2-ECD but not hFLRT2-ICD (**Figure 5E**). To address whether binding of UNC5B to the FLRT2 ECD is required for FLRT2′s role in instigating monocyte-to-macrophage differentiation, we transfected THP-1 cells with control, hFLRT2-ECD, or hFLRT2-ICD vector. As shown in **Figures 5F, G**, hFLRT2-ECD but not hFLRT2-ICD significantly promoted the differentiation of monocytes into macrophages, which was concomitant with a substantial increase in the mRNA and protein levels of macrophage markers. Together, our data indicated that FLRT2 directly interacts with UNC5B through its ECD, leading to monocyte-to-macrophage differentiation.

### Akt/mTOR signaling is involved in FLRT2-mediated THP-1 cell differentiation

**Figure 6:**
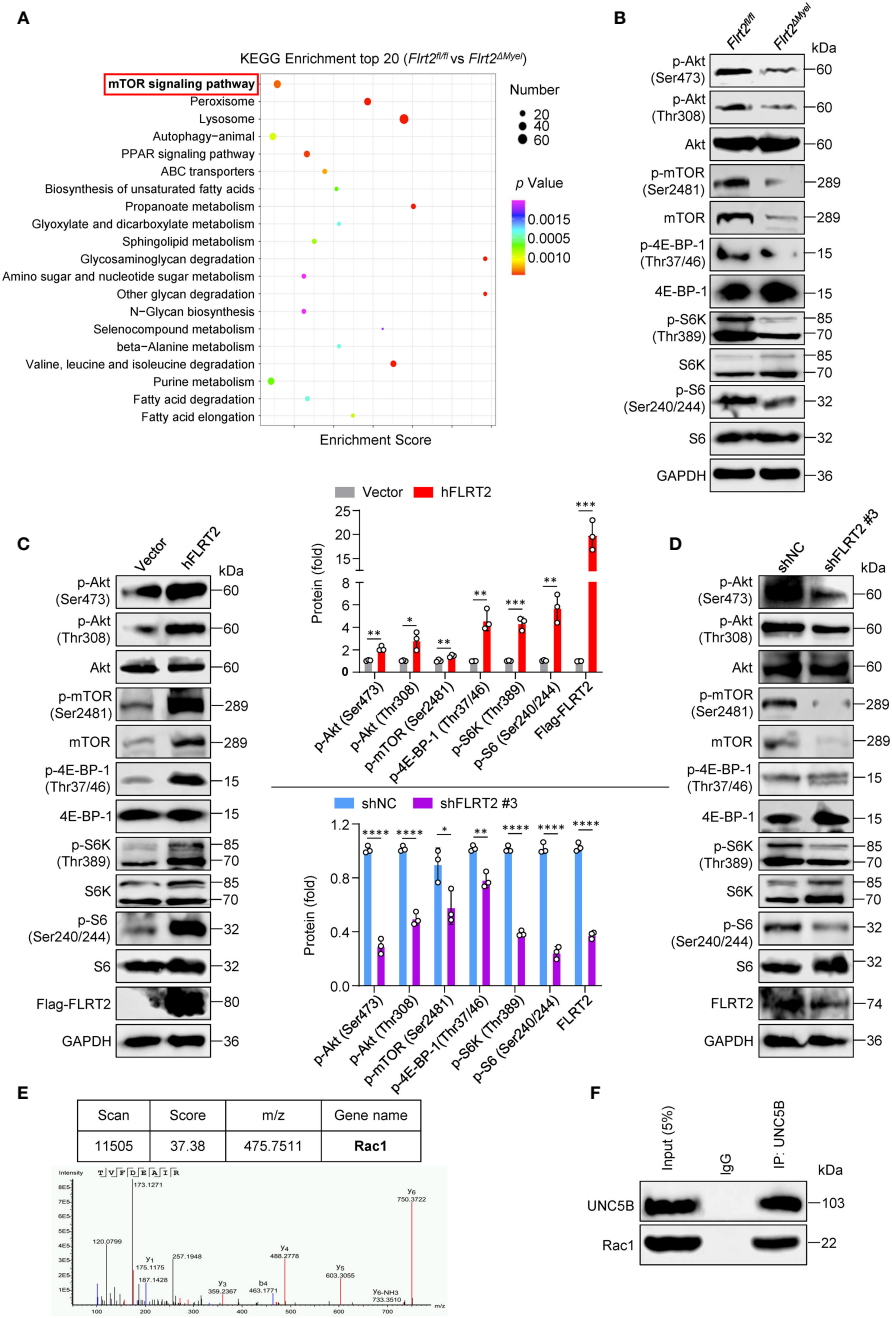
FLRT2 activates Akt/mTOR signaling *via* the binding of UNC5B with Rac1. **(A)** Kyoto Encyclopedia of Genes and Genomes (KEGG) pathway analysis of the upregulated differentially expressed genes (DEGs) in PMs of *Flrt2^fl/fl^* and *Flrt2^ΔMyel^* mice. **(B)** Immunoblot analysis of p-Akt (Ser473), p-Akt (Thr308), Akt, p-mTOR (Ser2481), mTOR, p-4E-BP-1 (Thr37/46), 4E-BP-1, p-S6K (Thr389), S6K, p-S6 (Ser240/244), and S6 proteins in PMs isolated from *Flrt2^fl/fl^* and *Flrt2^ΔMyel^* mice i.p. injected with thioglycollate for 3 days (n = 3 mice per group). **(C)** Immunoblot analysis of p-Akt (Ser473), p-Akt (Thr308), Akt, p-mTOR (Ser2481), mTOR, p-4E-BP-1 (Thr37/46), 4E-BP-1, p-S6K (Thr389), S6K, p-S6 (Ser240/244), S6, Flag-FLRT2, and FLRT2 protein levels in THP-1 cells transfected with control or hFLRT2 vector for 48 h (n = 3). **(D)** Immunoblot analysis of the indicated protein levels in THP-1 cells transfected with shNC or shFLRT2 #3 vector for 48 h (n = 3). **(E)** Immunoprecipitation samples were collected using anti-Flag antibody from control vector and Flag-UNC5B-overexpressing HEK293T cells and subjected to mass spectrometry (MS) to identify potential binding partners of UNC5B protein. A unique peptide of Rac1 protein was identified in the UNC5B-overexpressing immunoprecipitation samples by analyzing the mass-to-charge ratio of the samples. **(F)** The endogenous interaction between Rac1 and UNC5B was confirmed in THP-1 cells by co-immunoprecipitation (co-IP) analysis. Data are means ± SD. *P* values were determined using unpaired, two-tailed Student’s *t*-tests. ^*^ *P* < 0.05, ^**^ *P* < 0.01, ^***^ *P* < 0.001, ^****^ *P* < 0.0001.

To explore signaling mechanisms that underlie FLRT2-induced monocyte-to-macrophage differentiation, we applied RNA-seq analysis. Kyoto Encyclopedia of Genes and Genomes (KEGG) pathway enrichment analysis showed that one of the dominantly enriched pathways is mTOR signaling (**Figure 6A**). Zhao et al. have recently demonstrated that mTOR intrinsically promotes monocyte-to-macrophage differentiation (41). PI3K/Akt signaling also positively regulates monocyte differentiation into macrophages (42, 43). So, we hypothesized that FLRT2 might affect monocyte/macrophage development *via* the activation of Akt/mTOR pathway. Immunoblot analysis verified the inhibition of Akt/mTOR signaling in PMs of *Flrt2^ΔMyel^* mice versus *Flrt2^fl/fl^* littermates, as evidenced by the decreased levels of p-Akt (Ser473), p-Akt (Thr308), p-mTOR (Ser2481), p-4E-BP-1 (Thr37/46), p-S6K (Thr389), and p-S6 (Ser240/244) (**Figure 6B** and **Supplementary Figure 8**). Additionally, gain- and loss-of-function studies supported that FLRT2 positively modulated Akt/mTOR signaling in THP-1 cells (**Figures 6C, D**). To dissect how FLRT2 induces Akt/mTOR activation, we sought to identify a protein that lies upstream of the Akt/mTOR signaling pathway and can bind to UNC5B. In Flag-UNC5B-overexpressed HEK293T cells, cell lysates were immunoprecipitated by anti-Flag antibody and subjected to mass spectrometry (MS)-based analysis. This method identified 421 proteins bound to UNC5B (**Supplementary Figure 9A**). Crossing the identified proteins using MS analysis with proteins involved in the PI3K-Akt signaling pathway, a Venn diagram showed 6 overlapped proteins, including EIF4E, RPS6, PPP2R2D, Rac1, GNB3, and CDC37 (**Supplementary Figure 9A**). MS data of overlapping 6 proteins were listed in **Supplementary Figure 9B**. Rac1 was one of the binding proteins of UNC5B (**Figure 6E**). Because the listed top 3 proteins, including EIF4E, RPS6, and PPP2R2D, are located downstream of the PIK3/Akt signaling pathway, Rac1, an upstream of this cascade, was subjected to protein-protein interaction (PPI) analysis. The results showed the interaction between UNC5B and Rac1 (**Supplementary Figure 9C**). The co-IP experiments confirmed the direct interaction between Rac1 and UNC5B (**Figure 6F**). These results suggest that FLRT2 induces Akt/mTOR activation through the interaction of UNC5B and Rac1.

To address whether FLRT2-mediated THP-1 cell differentiation into macrophages requires PI3K/Akt/mTOR signaling, we treated FLRT2-overexpressed THP-1 cells with PI3K inhibitor LY-294002 or mTOR inhibitor rapamycin, respectively. Inhibition of these pathways had blocked FLRT2-induced differentiation of monocytes into macrophages, evidenced by decreased macrophage marker mRNA and protein levels and the percentage of THP-1-derived macrophages (**Supplementary Figures 10A–C**). Collectively, these results suggest that Akt/mTOR signaling is critically involved in FLRT2-promoted monocyte-to-macrophage differentiation.

### MHY1485, an mTOR agonist, abolished impaired peritoneal macrophage generation and functions in *Flrt2^ΔMyel^* mice

**Figure 7:**
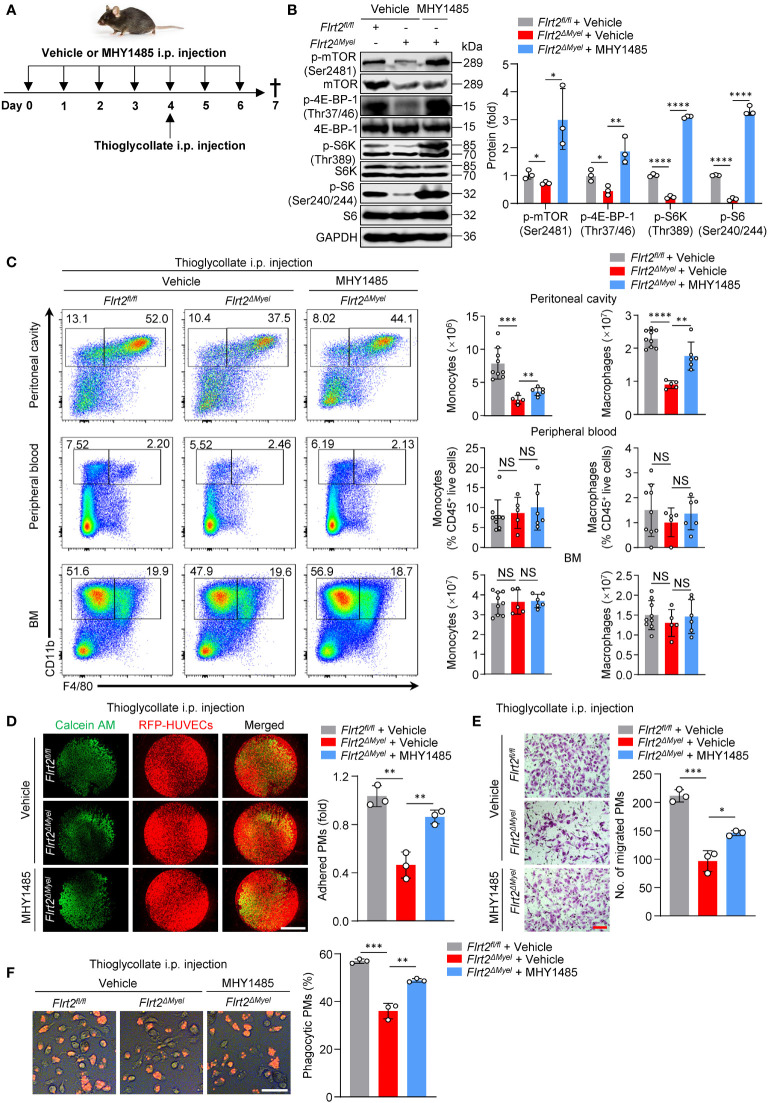
MHY1485, an mTOR agonist, abrogates abnormal peritoneal monocyte-macrophage differentiation and macrophage functions of *Flrt2^ΔMyel^* mice *in vivo*. **(A)** Protocol for *in vivo* rescue experiments. *Flrt2^fl/fl^* and *Flrt2^ΔMyel^* mice were infused with vehicle or mTOR activator MYH1485 (0.25 mg/kg) for 7 successive days by i.p. injection and were injected i.p. with thioglycollate at day 4. At the end of day 7, samples were collected for flow cytometric and functional analyses. **(B)** Immunoblot analysis of p-mTOR (Ser2481), mTOR, p-4E-BP-1 (Thr37/46), 4E-BP-1, p-S6K (Thr389), S6K, p-S6 (Ser240/244), and S6 levels in PMs isolated from *Flrt2^fl/fl^* and *Flrt2^ΔMyel^* mice (n = 3 mice per group). **(C)** Representative flow cytometric profiles and data plots showing the absolute numbers of CD45^+^CD11b^+^F4/80^−^ monocytes and CD45^+^CD11b^+^F4/80^+^ macrophages in the peritoneal cavity and BM, and the frequencies of CD45^+^CD11b^+^F4/80^−^ monocytes and CD45^+^CD11b^+^F4/80^+^ macrophages in peripheral blood of *Flrt2^fl/fl^* and *Flrt2^ΔMyel^* mice (n = 5, 6, or 9 mice per group). **(D)** At the end of day 7, PMs were collected from the above treated *Flrt2^fl/fl^* and *Flrt2^ΔMyel^* mice and then co-cultured with HUVECs expressing RFP for 6 h, followed by fluorescent microscopy (n = 3 mice per group). Scale bar, 5 mm. **(E)** At the end of day 7, PMs were isolated from the above treated *Flrt2^fl/fl^* and *Flrt2^ΔMyel^* mice and seeded in Boyden chambers for 48 h, followed by crystal violet staining (n = 3 mice per group). Scale bar, 100 μm. **(F)** At the end of day 7, zymosan was injected i.p. into the above treated *Flrt2^fl/fl^* and *Flrt2^ΔMyel^* mice for 2 h. PMs were collected and placed in a 6-well plate for 6 h. The phagocytic particles were observed under a laser confocal scanning microscope (n = 3 mice per group). Scale bar, 50 μm. Data are means ± SD. *P* values were determined using unpaired, two-tailed Student’s *t*-tests. NS, not significant. ^*^ *P* < 0.05, ^**^ *p* < 0.01, ^***^ *P* < 0.001, ^****^ *P* < 0.0001.

Our final question was whether mTOR activation could reverse the observed phenotypes in *Flrt2^ΔMyel^* mice. *Flrt2^fl/fl^* and *Flrt2^ΔMyel^* mice were administered as illustrated in **Figure 7A**. The reactivation of the mTOR signaling cascade was demonstrated by a significant increase in p-mTOR (Ser2481), p-4E-BP-1 (Thr37/46), p-S6K (Thr389), and p-S6 (Ser240/244) protein levels in PMs of *Flrt2^ΔMyel^* mice after MHY1485 administration (**Figure 7B**). Flow cytometry analysis showed FLRT2 loss caused a marked decrease in the number of PMs in *Flrt2^ΔMyel^* mice compared with *Flrt2^fl/fl^* mice (**Figure 7C**). MHY1485 rescued the reduction of CD11b^+^F4/80^+^ macrophage populations in the peritoneal cavity of *Flrt2^ΔMyel^* mice (**Figure 7C**). Consistently, functional assays demonstrated that MHY1485 reversed the impaired ability of adhesion, migration, and phagocytosis of FLRT2-null PMs (**Figures 7D–F**). Collectively, activation of mTOR signaling cascades by MHY1485 abolished inhibited peritoneal macrophage development and function in *Flrt2^ΔMyel^* mice.

To explore whether mTOR activator MHY1485 rescues the impaired function of human PBMCs with FLRT2 deficiency, we transfected PBMCs with shNC or shFLRT2 and treated them with MHY1485. In **Supplementary Figure 11A**, the results demonstrated that FLRT2 loss-induced mTOR inhibition was re-activated by MHY1485, evidenced by increased ratios of p-mTOR (Ser2481) to mTOR, p-4E-BP-1 (Thr37/46) to 4E-BP-1, p-S6K (Thr389) to S6K, and p-S6 (Ser240/244) to S6. Consistently, functional assays demonstrated that MHY1485 reversed the impaired ability of adhesion, migration, and phagocytosis of FLRT2-knockdown M-CSF-primed PBMC-derived macrophages (**Supplementary Figures 11B–D**). Collectively, the activation of mTOR signaling cascades by MHY1485 improves the impaired function of PBMCs with FLRT2 loss.

## Discussion

**Figure 8:**
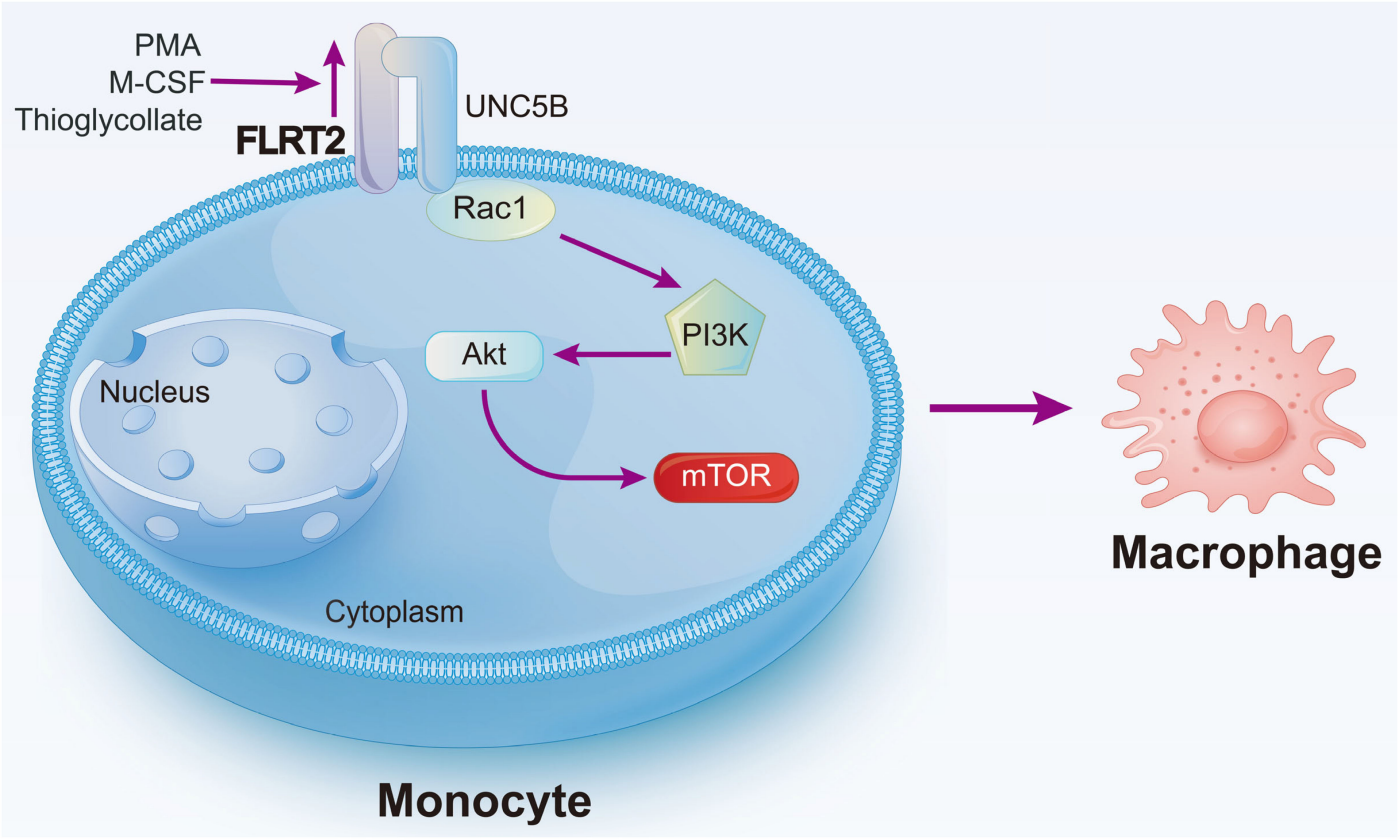
Schematic diagram of our major findings. Pro-differentiation factors, such as PMA, M-CSF, and thioglycollate, upregulate *Flrt2* expression in monocytes. FLRT2, in turn, accelerates monocyte-to-macrophage differentiation by binding to UNC5B *via* its ECD and subsequently activating the Akt/mTOR signaling pathway.

In this work, we have identified FLRT2 as an essential endogenous regulator of monocyte-to-macrophage differentiation. The relevance of this phenotype was confirmed using *Flrt2^ΔMyel^* mice *in vivo*. We also revealed that FLRT2 ECD binding to the receptor UNC5B leads to activation of the Akt/mTOR signaling pathway and consequent monocyte/macrophage development (**Figure 8**).

Monocytes are derived from MDPs in the BM through the common monocyte progenitors (cMoPs) stage (44–46). However, a recent study has challenged this classical model of hematopoiesis by using a fluorescent fate-mapping system to find that MDPs produce monocytes directly, not through cMoPs (47, 48). After being released into the blood, mouse monocytes are categorized into two main subsets, Ly6C^hi^ classical and Ly6C^low^ non-classical monocytes, equivalent to human CD14^+^ and CD14^low^CD16^+^ monocyte subsets, respectively (49). Ly6C^hi^ classical monocytes receive chemokine signals and migrate into inflamed or impaired tissues, where they give rise to dendritic cells (DCs) and macrophages (50). Ly6C^low^ non-classical monocytes monitor the endothelial surface and repair injured endothelium by recruiting neutrophils as needed (51, 52). However, the exact mechanisms underlying the differentiation of monocytes into macrophages are not completely understood. To the best of our knowledge, we have, for the first time, demonstrated that the endogenous protein FLRT2 drives human and mouse monocyte differentiation into macrophages. Importantly, we discovered that myeloid cell-specific FLRT2 deficiency blocks peritoneal macrophage generation *in vivo*. In seeking endogenous regulators of monocyte/macrophage development, Goudot et al. have recently identified three molecules, including interferon regulatory factor (IRF), MAF BZIP transcription factor B (MAFB), and AHR (12). They reported that the transcription factors, IRF4 and MAFB, regulate monocyte-to-dendritic cell and monocyte-to-macrophage differentiation, respectively (12). AHR functions as a molecular switch that drives monocyte-to-DC events while limits monocyte-to-macrophage differentiation (12). Since DCs share the same precursor cells with macrophages in BM, whether FLRT2 plays a role in the differentiation of monocytes into DCs warrants further investigation. In addition, *in vivo* investigation of the vital roles of FLRT2-regulated myelopoiesis in disease models, such as *Listeria monocytogenes* infection or myeloid leukemia, is needed in our future study.

As a single-transmembrane protein with a short intracellular domain and large extracellular domain (14), FLRT2 binds to FGFR2 and activates downstream pathways, suggesting a role in craniofacial development (39). FLRT2 also interacts with UNC5D and functions as a chemorepellent for UNC5-positive neurons to modulate the migration of cortical neurons (17). A study has recently reported that FLRT2 inhibits endothelial cell migration towards type I collagen by acting on G protein-coupled receptor latrophilin 2 (LPHN2) (53). Our RNA-seq data showed that UNC5B is upregulated in differentiated THP-1-derived macrophages. More importantly, the direct interaction of FLRT2 with UNC5B was confirmed in our systems. In line with our findings, Tai-Nagara et al. have reported that endothelial FLRT2 maintains the normal organization of the placental labyrinth *via* interaction with UNC5B (16). However, the effects of FLRT2 on the migration of THP-1 and HUVECs are the opposite. We observed that FLRT2 promotes the migration of THP-1-derived macrophages. Conversely, they found that FLRT2 shows inhibitory effects on the migration of HUVECs (16). We also revealed that FLRT2 binds to UNC5B through its ECD. Since FLRT3 interacts with UNC5B through its leucine-rich repeat (LRR), a specific part located in FLRT3 ECD (21, 54), it is possible that FLRT2 interacts with UNC5B *via* LRR.

Our studies have highlighted an essential role of the Akt/mTOR signaling pathway in mediating FLRT2-induced monocyte differentiation into macrophages. During embryonic development of the nervous system, netrin-1 binds to UNC5B and induces the interaction of UNC5B with GTPase PIKE-L, consequently leading to PI3K/Akt pathway activation and enhanced neuronal survival (55). We found that FLRT2 functions as another ligand for UNC5B and activates PI3K/Akt signaling. Downstream of PI3K/Akt, mTOR signaling has recently been reported to promote the development of granulocyte-monocyte progenitors into monocytes/macrophages *via* upregulating CD115 in a signal transducer and activator of transcription (STAT)/IRF8-dependent manner (41). Additionally, the mTORC1-S6K1-Myc axis has been shown to be critical to terminal myeloid development (56). Another study revealed the positive association between mTORC1-induced anabolic metabolism and enhanced myelopoiesis (57). In our obversions of overactivated Akt/mTOR signaling by FLRT2 overexpression in THP-1 cells, PI3K or mTOR inhibition by pharmacological compounds markedly blocked FLRT2-driven differentiation of THP-1 monocytes into macrophages, suggesting that FLRT2-mediated monocyte-to-macrophage differentiation requires Akt/mTOR signaling. Additionally, we dissected the critical roles of FLRT2 in activating the Akt/mTOR pathway, which expanded our understanding of the regulatory mechanism of this important pathway.

FLRT2 is expressed differentially under distinct pathophysiological conditions. Originally, FLRT2 was reported to be expressed in the pancreas, skeletal muscle, brain, and heart (14). But recent transcriptome sequencing data showed that FLRT2 was expressed in alveolar macrophages (27) and PMs (58). To our surprise, FLRT2 was almost undetectable in THP-1 monocytes but significantly increased in PMA-induced differentiated THP-1 macrophages. This conclusion is supported by a recent study showing that receptor activator of nuclear factor-κB ligand (RANKL) upregulates the expression of FLRT2 in osteoclasts (specialized macrophages), thereby promoting osteoclast multinucleation/maturation (59). Previous reports also showed that hypermethylation of the FLRT2 promoter led to decreased FLRT2 gene expression, which was involved in the development of prostate, breast, and colorectal cancers (23, 60, 61). FLRT2 gene expression is regulated by miRNA and is associated with the pathogenesis of giant cell bone tumor (62). Deciphering the regulators of FLRT2 is of great significance to further understand and modulate the functions of FLRT2.

## Conclusions

We report that FLRT2 drives monocyte differentiation into macrophages through binding to UNC5B and activating Akt/mTOR signaling. By deciphering a novel role for the endogenous membrane protein FLRT2 in promoting monocyte-to-macrophage differentiation, our findings may help develop therapeutic strategies for human diseases with dysregulated monocyte/macrophage differentiation, such as acute myeloid leukemia (AML).

## Data availability statement

The datasets presented in this study can be found in online repositories. The names of the repository/repositories and accession number(s) can be found below: PRJNA737351 and PRJNA763032 (SRA), PXD040319 (ProteomeXchange).

## Ethics statement

All mouse experiments in this study were approved by the Experimental Animal Center of Guangzhou Medical University (protocol no. 2019-143) and performed according to relevant ethical regulations.

## Author contributions

XD and YH conceived, designed, and supervised the study. YF, KM, and Y-MH carried out experiments, and analyzed and interpreted the data. XD assisted with mouse studies. YD provided technical support. XD made the figures and wrote the manuscript. KM, ZL, and X-LZ critically reviewed and revised the manuscript. YX and XY provided intellectual input. All authors have read and approved the final submitted manuscript.
