# Supplementary material for: Fibronectin leucine-rich transmembrane protein 2 drives monocyte differentiation into macrophages via the UNC5B-Akt/mTOR axis
Source: Front Immunol. 2023 Apr 6;14:1162004. doi: 10.3389/fimmu.2023.1162004 (PMC10117657; doi:10.3389/fimmu.2023.1162004)
Supplement: Supplementary file 1 [file DataSheet_1.pdf]

Supplementary material

Fibronectin leucine-rich transmembrane protein 2 (FLRT2) drives monocyte differentiation into macrophages via the UNC5B-Akt/mTOR axis

Yaxiong Fang, Kongyang Ma, Yi-Min Huang, Yuanye Dang, Zhaoyu Liu, Yiming Xu, Xi-Long Zheng, Xiangdong Yang, Yongliang Huo, Xiaoyan Dai

The PDF file includes:

Supplementary Tables 1 to 3

Supplementary Figures 1 to 11

Supplementary Table 1. Key resources.

REAGENT or RESOURCE	SOURCE	IDENTIFIER
Antibodies		
Rat monoclonal anti-CD16/32 (FCM)	Biolegend	Cat# 101320; RRID: AB_1574973
Rat monoclonal anti-CD45 (FCM)	Biolegend	Cat# 101326; RRID: AB_4935535
Rat monoclonal anti-F4/80 (FCM)	Invitrogen	Cat# 17-4801-80; RRID: AB_2784647
Rat monoclonal anti-CD11b (FCM)	Biolegend	Cat# 101206; RRID: AB_312789
Rat monoclonal anti-CD19 (FCM)	Biolegend	Cat# 115529; RRID: AB_830706
Goat polyclonal anti-FLRT2 (WB and IF)	R&D	Cat# AF2877; RRID: AB_2106600
Rabbit polyclonal anti-FLRT2 (FACS)	Abcam	Cat# ab154023
Rabbit polyclonal anti-UNC5B (WB, IP, and IF)	Biorbyt	Cat# orb542246
Mouse monoclonal anti-β-Actin (WB)	Santa Cruz	Cat# sc-47778; RRID: AB_2714189
Mouse monoclonal anti-GAPDH (WB)	Santa Cruz	Cat# sc-137179; RRID: AB_2232048
Mouse monoclonal anti-CD36 (WB)	Santa Cruz	Cat# sc-7309; RRID: AB_627044
Goat polyclonal anti-SR-A (WB)	R&D	Cat# AF2708; RRID: AB_663950
Chicken polyclonal anti-GFP (IF)	Abcam	Cat# ab13970; RRID: AB_300798

Rabbit polyclonal anti-Myc (WB & IP)	Proteintech	Cat# 16286-1-AP; RRID: AB_11182162
Rabbit polyclonal anti-FLAG (WB)	Invitrogen	Cat# PA1-984B; RRID: AB_347227
Mouse monoclonal anti-FLAG (WB)	Proteintech	Cat# 66008-3-Ig; RRID: AB_2749837
Rabbit polyclonal anti-Akt (WB)	Cell Signaling Technology	Cat# 9272; RRID: AB_329827
Rabbit polyclonal anti-Phospho-Akt (Ser473) (WB)	Cell Signaling Technology	Cat# 9271; RRID: AB_329825
Rabbit monoclonal anti-Phospho-Akt (Thr308) (WB)	Cell Signaling Technology	Cat# 4056; RRID: AB_331163
Rabbit monoclonal anti-mTOR (WB)	Cell Signaling Technology	Cat# 2983; RRID: AB_2105622
Rabbit monoclonal anti-4E-BP1 (WB)	Cell Signaling Technology	Cat# 9644; RRID: AB_2097841
Rabbit monoclonal anti-Phospho-4E-BP1 (Thr37/46) (WB)	Cell Signaling Technology	Cat# 2855; RRID: AB_560835
Rabbit polyclonal anti-S6K (WB)	Cell Signaling Technology	Cat# 9202; RRID: AB_331676
Rabbit monoclonal anti-Phospho-S6K (Thr389) (WB)	Cell Signaling Technology	Cat# 9234; RRID: AB_2269803
Rabbit monoclonal anti-S6 (WB)	Cell Signaling Technology	Cat# 2217; RRID: AB_331355
Mouse monoclonal anti-Rac1(WB)	Santa Cruz	Cat# sc-514583; RRID: AB_2818941
Normal Rabbit Control IgG	Sino Biological	Cat# CR1
Rabbit polyclonal anti-	Cell Signaling	Cat# 2974; RRID: AB_2262884

Phospho- mTOR (Ser2481) (WB)	Technology	
Rabbit polyclonal anti-CD11B (WB)	Beyotime	Cat# AF6396
Bacterial and Virus Strains		
Stbl3 Chemically Competent Cell	Kangti Life Technology Co., Ltd.	Cat# KTSM110L
RFP overexpression lentivirus	This paper	N/A
Chemicals		
Zombie Aqua™ Fixable Viability Kit	BioLegend	Cat# 423101
Anti-FLAG® M2 Magnetic Beads	Sigma-Aldrich	Cat# M8823
Protein A/G Magnetic Beads	MedChemExpress	Cat# HY-K0202
Phorbol 12-myristate 13-acetate	Sigma-Aldrich	Cat# P8139; CAS: 16561-29-8
Dimethyl sulfoxide	Sigma-Aldrich	Cat# D2650; CAS: 67-68-5
Crystal Violet	Sigma-Aldrich	Cat# C0775; CAS: 548-62-9
Zymosan A (S. cerevisiae) BioParticles™	Invitrogen	Cat# Z23374
LY-294,002 hydrochloride	Sigma-Aldrich	Cat# L9908; CAS: 934389-88-5
Rapamycin	Sigma-Aldrich	Cat# V900930; CAS: 53123-88-9
Calcein AM	Beyotime	Cat# C2012; CAS: 148504-34-1
MHY1485	Sigma-Aldrich	Cat# SML0810; CAS: 326914-06-1
DAPI	Invitrogen	Cat# D1306; CAS: 28718-90-3
Recombinant human M-CSF protein	R&D	Cat# 216-MCC

Critical Commercial Assays		
qPCR	Accurate Biology	Cat# AG11701
Experimental Models: Cell Lines		
Human: HEK239T	ATCC	Cat# CRL-3216
Human: HUVEC	ATCC	Cat# PCS-100-013
Human: Peripheral blood mononuclear cell	LDEBIO	Cat# 1501-50M
Experimental Models: Organisms		
Mouse: C57BL/6J	Guangdong Medical Laboratory Animal Center	N/A
Mouse: B6.129P2-*Lyz2*$^{tm1(cre)Ifo/J}$	The Jackson Laboratory	Cat# JAX: 004781
Mouse: C57BL/6-*Flrt2*$^{em1(flox)Smoc}$	This paper	N/A
Oligonucleotides		
shRNA targeting sequence: hFLRT2 #1: 5'-CATCTGATCAGGCTCTATTTGCTC-3'	This paper	N/A
shRNA targeting sequence: hFLRT2 #2: 5'-GTCTCCTTAAATAACGATC	This paper	N/A

AACTC-3'		
shRNA targeting sequence: hFLRT2 #3: 5'-CGTCAGGGAATTAAATATGAACTC-3'	This paper	N/A
shRNA targeting sequence: hUNC5B #1: 5'-AGGTGGAATGGCTCAAGAATGAG-3'	This paper	N/A
shRNA targeting sequence: hUNC5B #2: 5'-AGGAGCCGAAACCGCTAATGTTCA-3'	This paper	N/A
shRNA targeting sequence: hUNC5B #3: 5'-TGTCGGACACTGCCAACTATAC-3'	This paper	N/A
shRNA targeting sequence: negative control 5'-CGTACGCGGAATACTTCGA-3'	This paper	N/A
Primers for plasmid construction, see **Supplementary Table 2**	This paper	N/A

Primers for qPCR, see **Supplementary Table 3**	This paper	N/A
Primers used to genotype *FLRT2*$^{fl/fl}$ mice:<br>F: 5'-TAGAGGTCCAGCGTTAGAAAG- 3';<br>R: 5'-TGAGCCCACCTGACATTATC- 3'	This paper	N/A
Primers used to genotype *Lyz2*^{Cre} mice:<br>F: 5'-AGCGATGGATTTCCGTCTCTGG- 3';<br>R: 5'-AGCTTGCATGATCTCCGGTATTGAA- 3'	This paper	N/A
Recombinant DNA		
pLvTHM-Venus (expression vector for GFP)	This study	N/A
pLvTHM-Venus-hFLRT2 (expression vector for recombinant human FLRT2)	This study	N/A
pLvTHM-mApple (expression vector for RFP)	This study	N/A
pLvTHM-Venus-hUNC5B (expression vector for recombinant human UNC5B)	This study	N/A

pWPI-3XFlag/Strep (expression vector for Flag)	This study	N/A
pWPI-3XFlag/Strep-hFLRT2 (expression vector for recombinant human FLRT2)	This study	N/A
pWPI-3XFlag/Strep-hFLRT2-ED (expression vector for recombinant human FLRT2-ED)	This study	N/A
pWPI-3XFlag/Strep-hFLRT2-ID (expression vector for recombinant human FLRT2-ID)	This study	N/A
pKMyc (expression vector for Myc)	This study	N/A
pKMyc-hUNC5B (expression vector for recombinant human UNC5B)	This study	N/A
Software		
ImageJ 1.51 s	N/A	https://imagej.nih.gov/ij/
GraphPad Prism 8	N/A	https://www.graphpad.com

Supplementary Table 2. Sequences of primers used for plasmid construction.

Name		Sequence 5'-3'
hFLRT2	Forward	ATGGGCCTACAGACCACAAAG
	Reverse	TCACGTATGGCAGTGCTCCAG
hUNC5B	Forward	ATGGGGGCCCGGAGCGGAGCT
	Reverse	TCAGCAGTCCCCGTCGGTGGC
hFLRT2-ECD	Forward	ATGGGCCTACAGACCACAAAG
	Reverse	TCAAAAGGGGGAGCCCATGCT
hFLRT2-ICD	Forward	ATGTGCTGGCATATGCACAAA
	Reverse	TCACGTATGGCAGTGCTCCAG

Supplementary Table 3. Sequences of the primers used for qPCR.

Gene	Species		Sequence
Flrt2	Homo	Forward	ACCACCCATGCCTCCTATCT
		Reverse	AAAGACGCTGAGCAAGACCA
Unc5b	Homo	Forward	ACTCACGGGGCAATGACTGG
		Reverse	AGGTCCAGGATCACACCCGT
Itgam	Homo	Forward	GCCGGTGAAATATGCTGTCT
		Reverse	GCGGTCCCATATGACAGTCT
Cd14	Homo	Forward	TCCCGGCCATCCAGAATCTA
		Reverse	AGCGAACGACAGATTGAGGG
Csf1r	Homo	Forward	TGTGGAGATGAGGCCTGTCT
		Reverse	TTCTTGGAAGCGAGGAAGGC
Msr1	Homo	Forward	TTGTGACGATCGCTGGGAAG
		Reverse	ATGTGAACAGGCTCTTGTCCC
Cd36	Homo	Forward	CAAAACGGCTGCAGGTCAAC
		Reverse	AAGCAACAAACATCACCACACC
Olr1	Homo	Forward	GCAGAAGAAGCTTCACAGGAGT
		Reverse	TTTCTCCATGCCAGATCCAGTC
Gapdh	Homo/Mus	Forward	CAAGGTCATCCATGACAACTTTG
		Reverse	GTCCACCACCCTGTTGCTGTAG
Flrt2	Mus	Forward	CTCTCTGTTCCAAGCCCCAG
		Reverse	ACGAGACTGTGGCCCATTTT
Unc5b	Mus	Forward	GGAACTACCAAGAGTCGCCG
		Reverse	TCAATGGTGAGCAGGAAGTTAGTG
Itgam	Mus	Forward	CATCTGCCAAGACGATCTCA
		Reverse	TTCTGGCTTGCTGAATCCTT
Cd14	Mus	Forward	GCTCAACTTTTCCTGCGAAC
		Reverse	CCCGCAGTGAATTGTGACTA
Csf1r	Mus	Forward	GACCTGCTCCACTTCTCCAG

		Reverse	GATGTCCCTAGCCAGTCCAA
Msr1	Mus	Forward	GGGCTTACTGGACAAACTGGT
		Reverse	CGCCTACACTCCCCTTCTCT
Cd36	Mus	Forward	TCGGAACTGTGGGCTCATTG
		Reverse	TCTTTGCCACGTCATCTGGG
Olr1	Mus	Forward	TTCCATGGGCCCTTTAGCTG
		Reverse	GTAAAGAAACGCCCCTGGTCT

Supplementary Figure 1

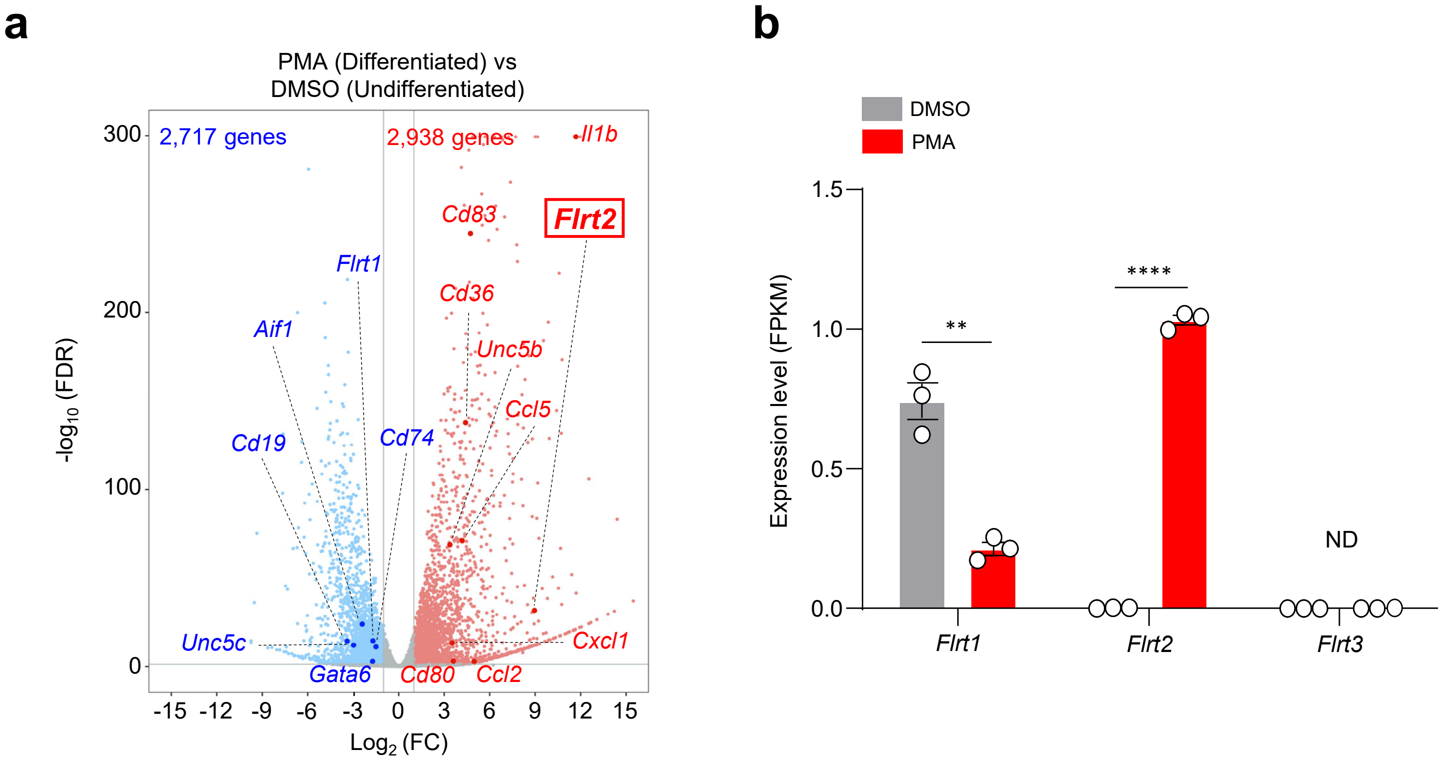

Supplementary Figure 1. FLRT2 is markedly upregulated in PMA-treated THP-1 cells. a Volcano plot of RNA-seq data showing differentially expressed genes (DEGs) in THP-1 cells treated with DMSO or PMA (100 ng/ml) for 24 h. Genes with substantially increased and decreased peak expressions are highlighted in red and blue, respectively. Biological replicates, n = 3. **b** RNA-seq analysis of *Flrt1*, *Flrt2*, and *Flrt3* expression in the cells. Data are means ± SD. *P* values were determined using unpaired, two-tailed Student's *t*-tests. ND, not detected.$^{**}P < 0.01$, $^{****}P < 0.0001$.

Supplementary Figure 2

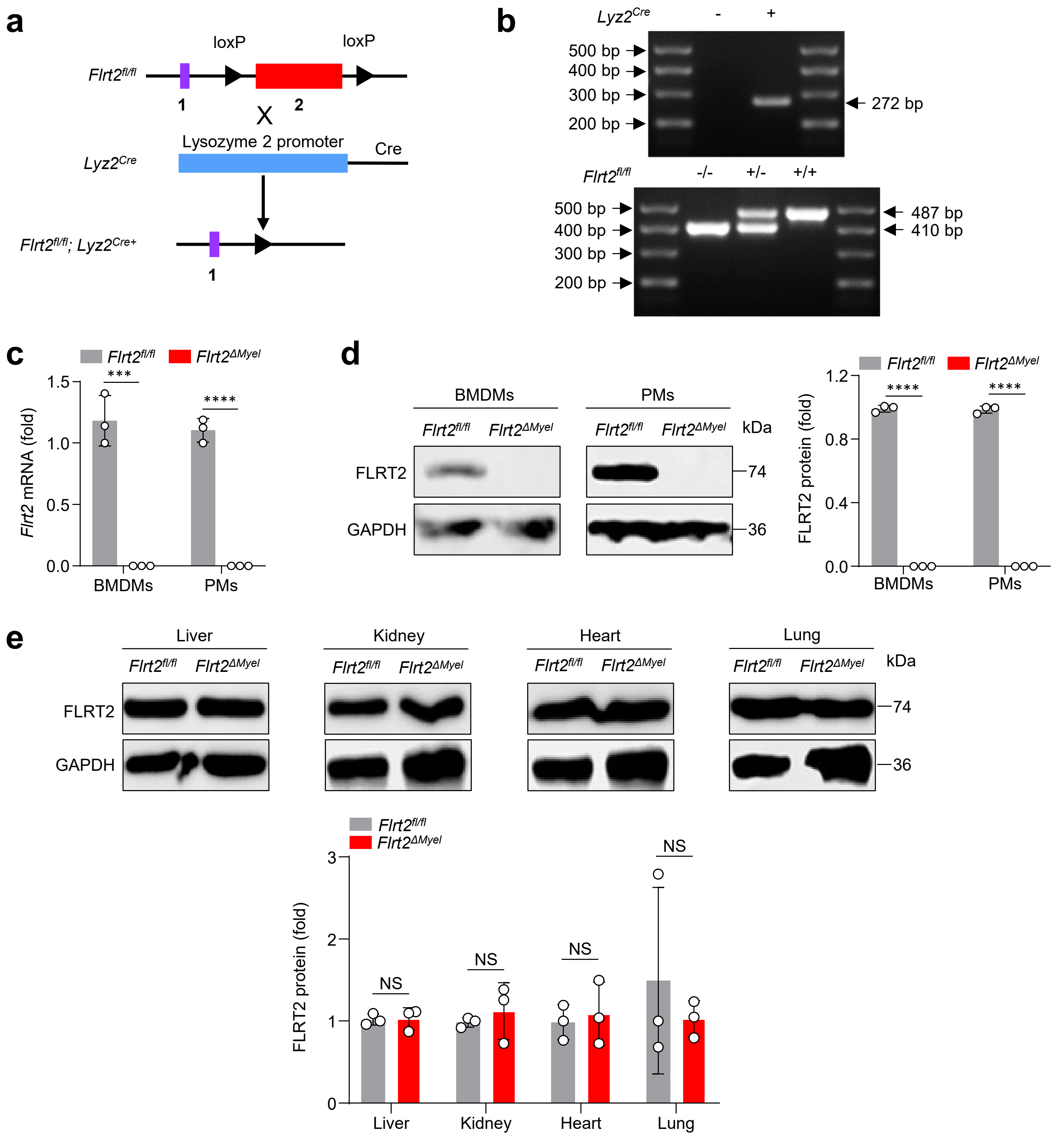

Supplementary Figure 2. Generation and identification of myeloid cell-specific FLRT2 knockout (*Flrt2*$^{\Delta Myel}$) mice. a Diagram illustrating how to generate *Flrt2*$^{\Delta Myel}$ mice. **b** Representative genotyping analysis of 3-week-old mice. *Flrt2*$^{fl/fl}$; *Lyz2*$^{Cre+}$ and *Flrt2*$^{fl/fl}$; *Lyz2*$^{Cre-}$ mice serve as *Flrt2*$^{\Delta Myel}$ and *Flrt2*$^{fl/fl}$ mice, respectively. **c** qPCR analysis of *Flrt2* mRNA in BMDMs and PMs isolated from *Flrt2*$^{fl/fl}$ and *Flrt2*$^{\Delta Myel}$ mice (n = 3 mice per group). **d, e** Immunoblot analysis of FLRT2 protein in bone marrow-derived macrophages (BMDMs), peritoneal macrophages (PMs), liver, kidney, heart, and lung isolated from *Flrt2*$^{fl/fl}$ and *Flrt2*$^{\Delta Myel}$ mice (n = 3 mice per group). Data are means ± SD. P values were determined using unpaired, two-tailed Student's t-tests. NS, not significant. $^{***}P < 0.001$, $^{****}P < 0.0001$.

Supplementary Figure 3

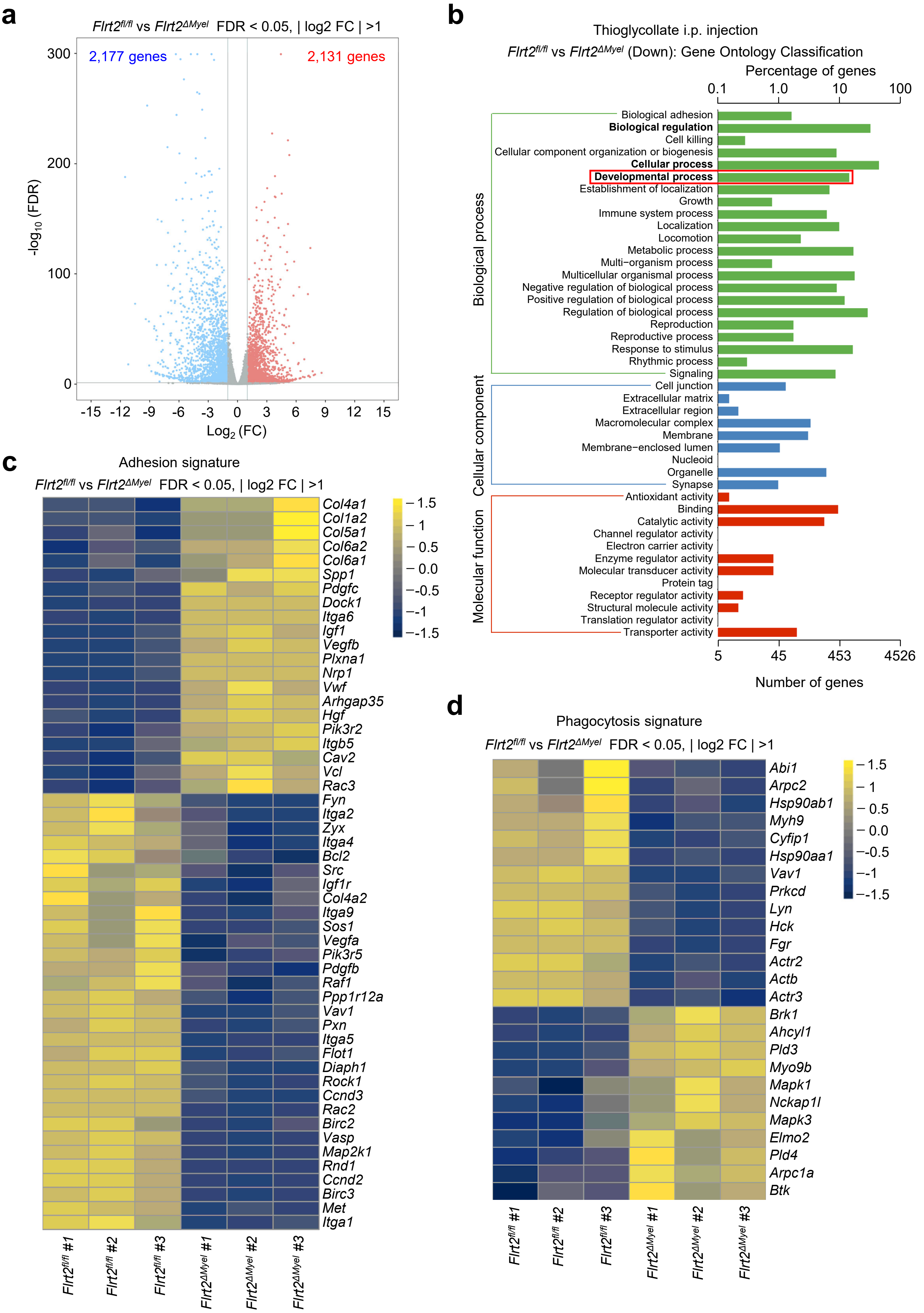

Supplementary Figure 3. RNA-seq analysis of peritoneal macrophages (PMs) of *Flrt2*$^{fl/fl}$ and *Flrt2*$^{\Delta Myel}$ mice. **a** PMs were isolated from *Flrt2*$^{fl/fl}$ and *Flrt2*$^{\Delta Myel}$ mice after 3 days of i.p. thioglycolate injection, and subjected to RNA-seq analysis. Volcano plots showing total differentially expressed genes (DEGs) in PMs (n = 3 mice per group). **b** Gene ontology classification of downregulated DEGs in PMs of *Flrt2*$^{\Delta Myel}$ mice in relative to *Flrt2*$^{fl/fl}$ mice. **c, d** Heat map showing dysregulated genes relevant to macrophage adhesion (**c**) and phagocytosis (**d**) in PMs of *Flrt2*$^{fl/fl}$ and *Flrt2*$^{\Delta Myel}$ mice (n = 3 mice per group).

Supplementary Figure 4

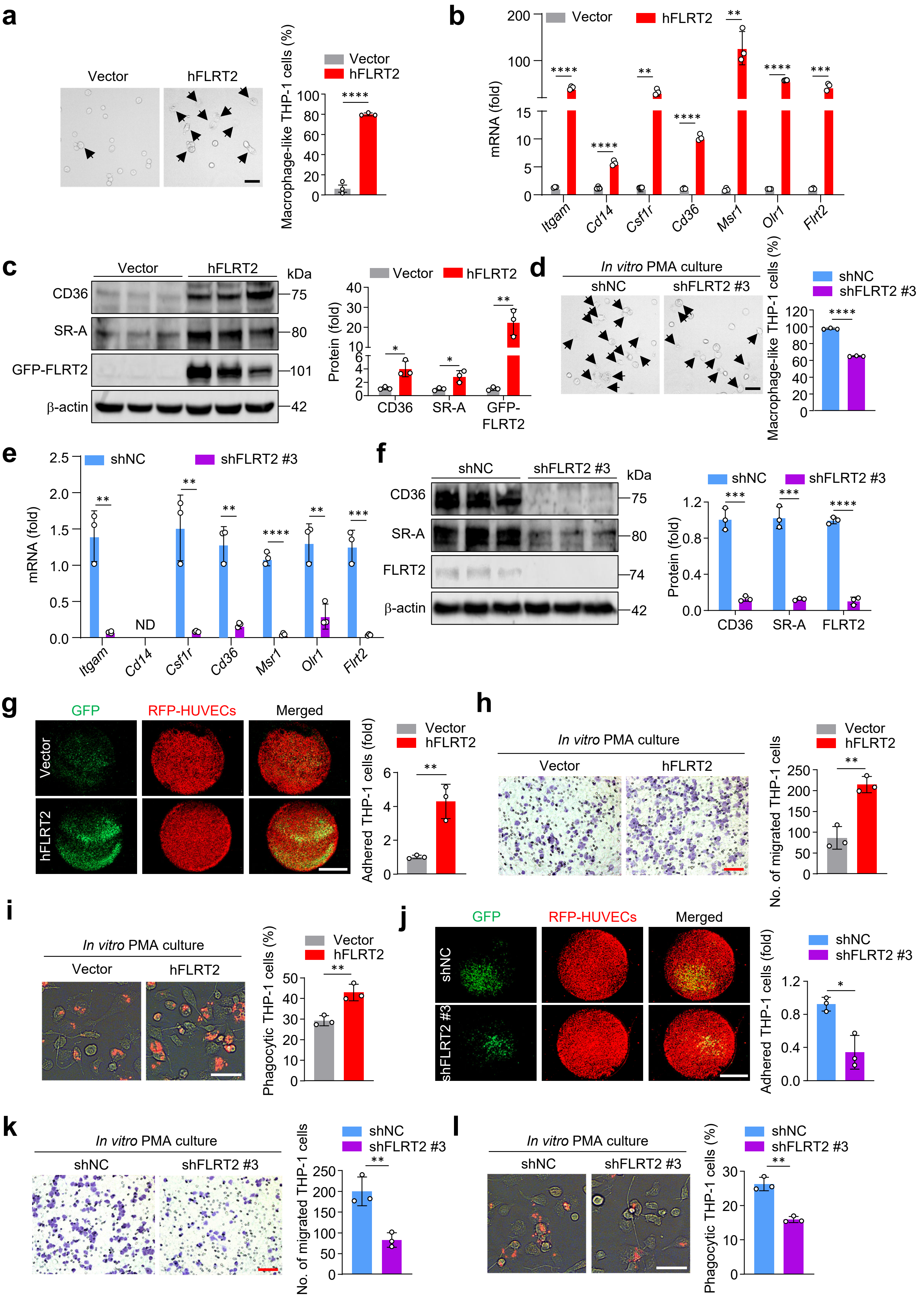

Supplementary Figure 4. FLRT2 promotes THP-1 monocyte differentiation into macrophages and enhances the adhesion, migration, and phagocytosis of differentiated THP-1-derived macrophages. a, d Representative phase-contrast light microscopy images showing morphological alterations of THP-1 cells transfected and treated as indicated (n = 3). Scale bar, 50 µm. **b, e** qPCR analysis of *Itgam*, *Cd14*, *Csf1r*, *Cd36*, *Msr1*, *Olr1*, and *Flrt2* mRNA in THP-1 cells transfected as indicated (n = 3). **c, f** Immunoblot analysis of CD36, SR-A, GFP-FLRT2, and FLRT2 proteins in THP-1 cells transfected as indicated (n = 3). **g** HUVECs expressing RFP were co-cultured with THP-1 cells expressing GFP or hFLRT2-GFP. After 6 h co-culture, pictures were taken using a fluorescence microscope (n = 3). Scale bar, 5 mm. **h, i** THP-1 cells were transfected with control or hFLRT2 vector for 24 h and treated with 100 ng/ml PMA for an additional 24 h. **h** Transwell cell migration assays were performed, and numbers of migrated cells were counted (n = 3). Scale bar, 100 µm. **i** Phagocytosis assay was performed by culturing the cells in Texas red-conjugated zymosan particles for 2 h at 37°C (n = 3). Cells were viewed for internalization of the particles by fluorescence microscopy. Scale bar, 50 µm. **j** HUVECs expressing RFP were co-cultured with THP-1 cells expressing GFP-shControl or GFP-shFLRT2 #3. After 6 h co-culture, pictures were taken with a fluorescence microscope (n = 3). Scale bar, 5 mm. **k, l** THP-1 cells were transfected with negative control shRNA (shNC) or FLRT2 targeting shRNA #3 (shFLRT2 #3) for 24 h and treated with 100 ng/ml PMA for an additional 24 h. **k** Migration assay was performed as described in (**h**). **l** Phagocytosis assay was performed as described in (**i**). Data are means ± SD. *P* values were determined using unpaired, two-tailed Student's *t*-tests. ND, not detected. $^{*}P < 0.05$, $^{**}P < 0.01$, $^{***}P < 0.001$, $^{****}P < 0.0001$.

Supplementary Figure 5

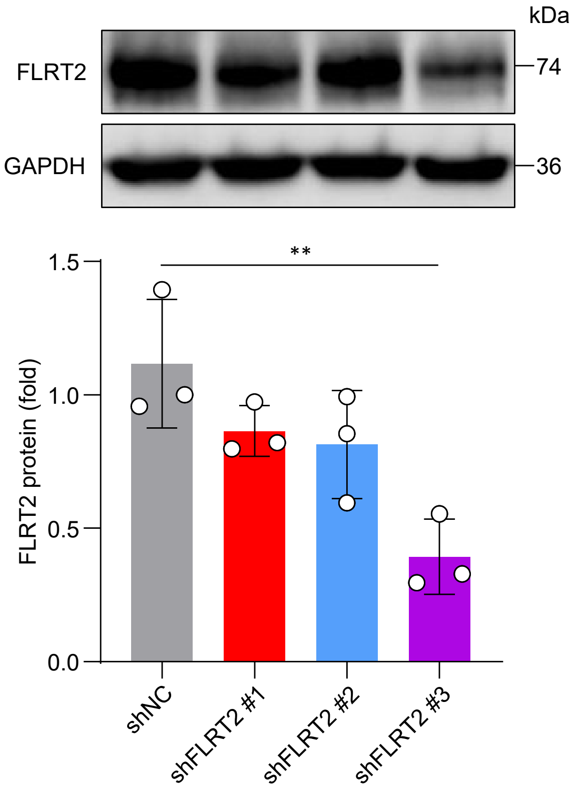

Supplementary Figure 5. FLRT2 is effectively silenced by shFLRT2 #3. Immunoblot analysis of FLRT2 protein of negative control shRNA (shNC) or FLRT2 targeting shRNA (shFLRT2)-transfected HEK293T cells (n = 3). Data are means ± SD. *P* values were determined using unpaired, two-tailed Student's *t*-tests. $^{**}P < 0.01$.

Supplementary Figure 6

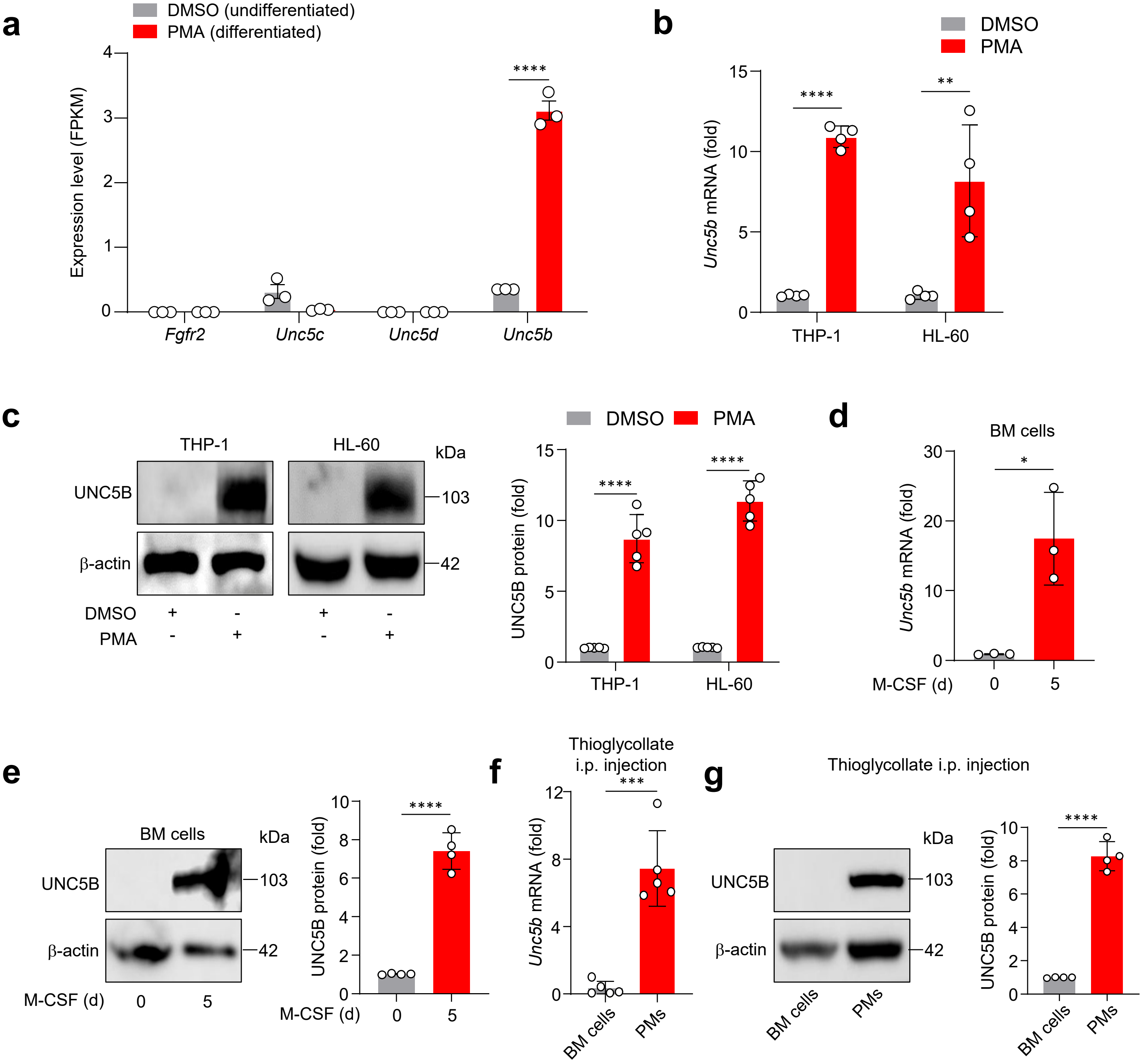

Supplementary Figure 6. UNC5B expression is significantly promoted during monocyte-to-macrophage differentiation. a RNA-sequencing analysis of *Fgfr2*, *Unc5c*, *Unc5d*, and *Unc5b* expression in DMSO- and PMA-treated THP-1 cells (n = 3). **b, c** qPCR analysis of *Unc5b* mRNA (**b**, n = 4) and immunoblot analysis of UNC5B protein (**c**, n = 5) in THP-1 or HL-60 treated with DMSO or PMA (100 ng/ml) for 24 h. **d, e** qPCR analysis of *Unc5b* mRNA (**d**, n = 3) and immunoblot analysis of UNC5B protein in BM cells induced by 25 ng/ml M-CSF for the indicated time (**e**, n = 4). **f, g** qPCR analysis of *Unc5b* mRNA (**f**, n = 5 mice per group) and immunoblot analysis of UNC5B protein (**g**, n = 4 mice per group) in BM cells and PMs isolated from C57BL/6J mice after 3 days of i.p. thioglycollate injection. Data are means ± SD. *P* values were determined using unpaired, two-tailed Student's *t*-tests. $^{*}P < 0.05$, $^{**}P < 0.01$, $^{***}P < 0.001$, $^{****}P < 0.0001$.

Supplementary Figure 7

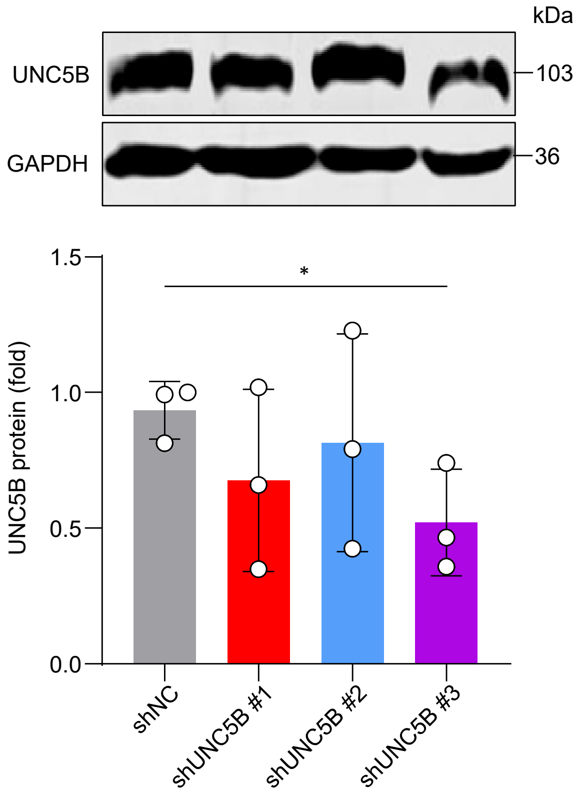

Supplementary Figure 7. UNC5B is effectively silenced by shUNC5B #3. Immunoblot analysis of FLRT2 protein of shNC or UNC5B targeting shRNA (shUNC5B)-transfected HEK293T cells (n = 3). Data are means ± SD. *P* values were determined using unpaired, two-tailed Student's *t*-tests. $^{*}P < 0.05$.

Supplementary Figure 8

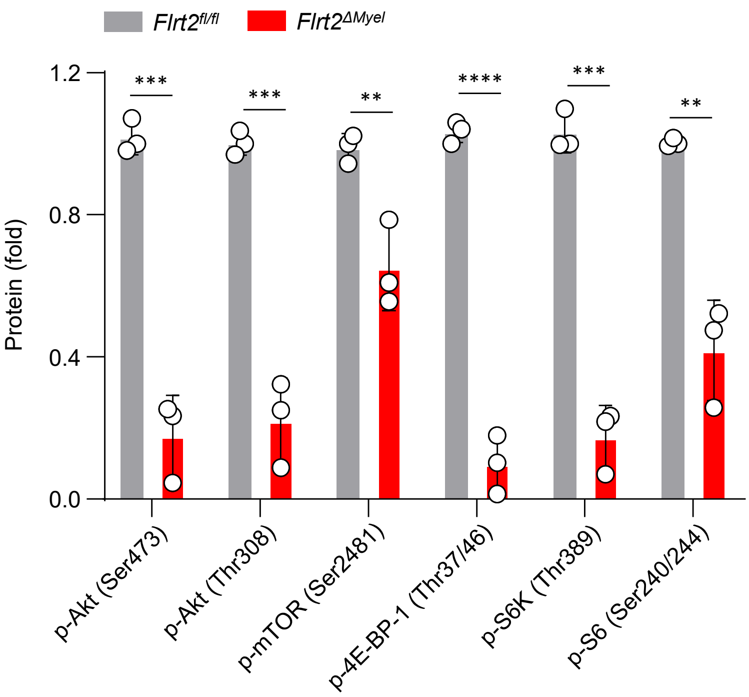

Supplementary Figure 8. Akt/mTOR pathway is inhibited in FLRT2-null PMs (related to main Figure 6b). Quantification of immunoblots of p-Akt (Ser473), p-Akt (Thr308), Akt, p-mTOR (Ser2481), mTOR, p-4E-BP-1 (Thr37/46), 4E-BP-1, p-S6K (Thr389), S6K, p-S6 (Ser240/244), and S6 proteins in PMs isolated from *Flrt2*$^{fl/fl}$ and *Flrt2*$^{\Delta Myel}$ mice i.p. injected with thioglycollate for 3 days (n = 3 mice per group). Data are means ± SD. *P* values were determined using unpaired, two-tailed Student's *t*-tests. $^{**}P < 0.01$, $^{***}P < 0.001$, $^{****}P < 0.0001$.

Supplementary Figure 9

a

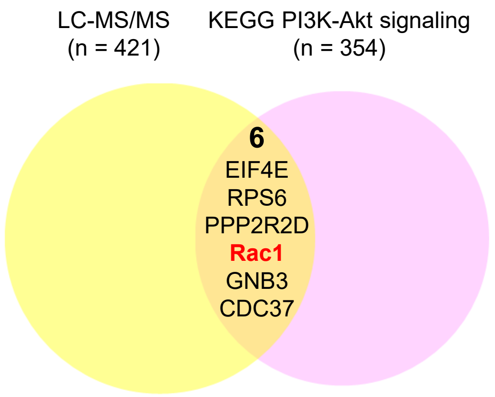

b

Protein	-10lgP	Coverage (%)	Coverage (%) EXP	#Peptides	#Unique	#Spec EXP
EIF4E	70.16	10	10	3	2	3
RPS6	71.43	7	7	4	3	4
PPP2R2D	52.07	4	4	2	2	2
**Rac1**	**48.26**	**4**	**4**	**2**	**1**	**2**
GNB3	34.26	3	3	1	1	2
CDC37	31.93	2	2	1	1	1

c

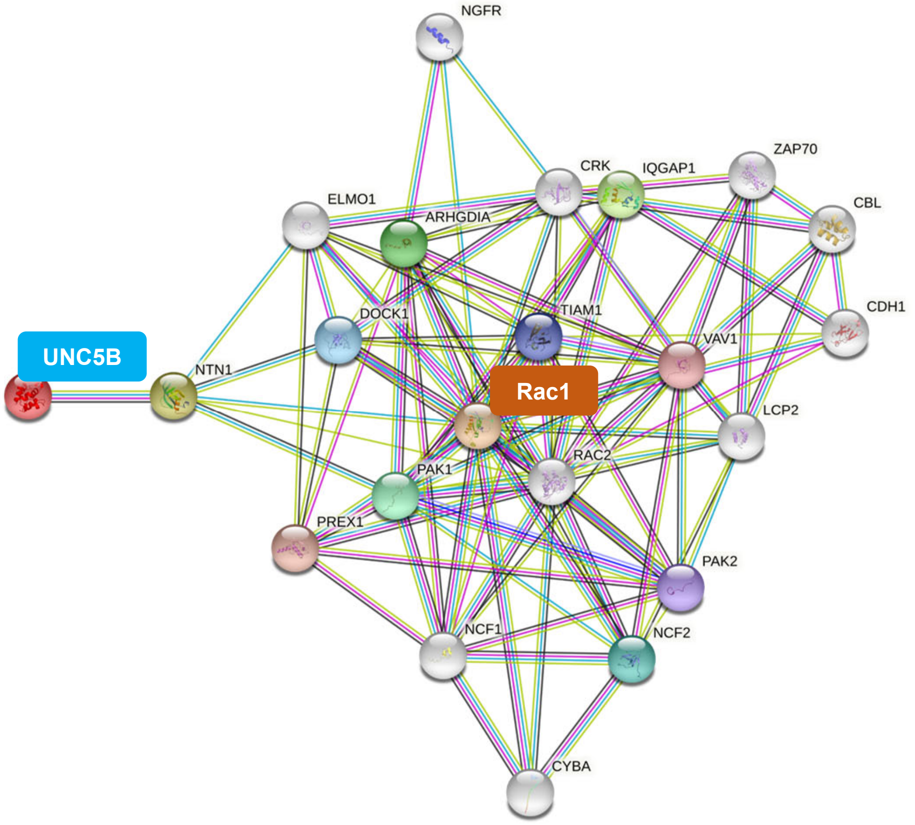

Supplementary Figure 9. UNC5B interacts with Rac1. a Venn diagram depicting the overlap of proteins identified by mass spectrometry associated with PI3K-Akt signaling pathway. **b** Mass spectrometry data of 6 overlapping proteins. **c** Protein-protein interaction (PPI) analysis was performed using the STRING database (STRING v11.0) (https://string-db.org/).

Supplementary Figure 10

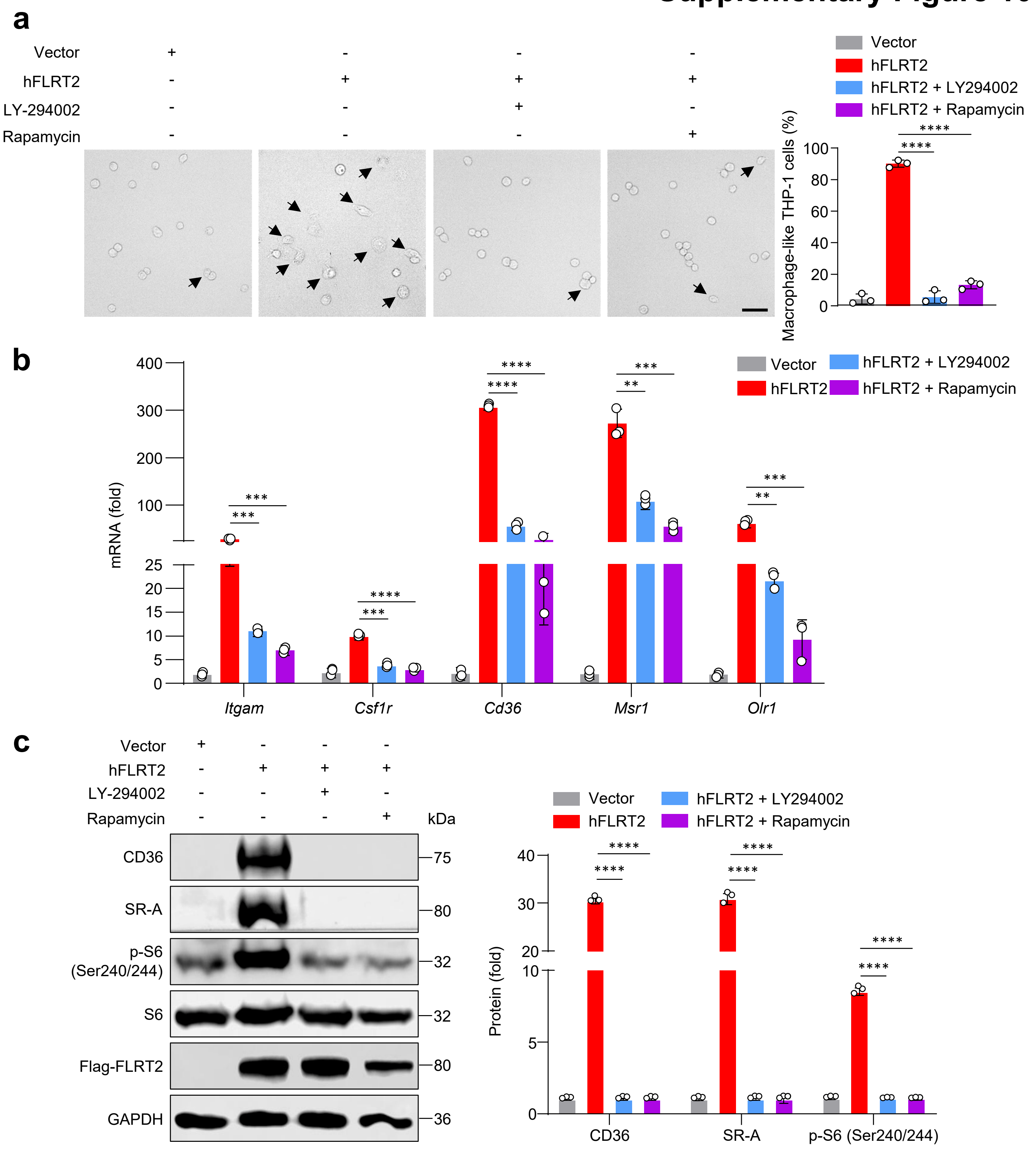

Supplementary Figure 10. Blocking PI3K/Akt/mTOR signaling negated FLRT2-triggered THP-1 cell differentiation towards macrophages. a–cTHP-1 cells were transfected with control or hFLRT2 vector for 48 h and cultured with 20 µM PI3K inhibitor LY-294002 for 12 h or 100 nM mTOR inhibitor rapamycin for 24 h. **a** Images were taken with a phase-contrast light microscope (n = 6). Scale bar, 50 µm. **b** *Itgam*, *Csf1r*, *Cd36*, *Msr1*, and *Olr1* mRNA levels in THP-1 cells were examined by qPCR (n = 3). **c** Cell lysates were collected and subjected to immunoblot analysis (n = 3). Data are means ± SD. *P* values were determined using unpaired, two-tailed Student's *t*-tests. $^{**}P < 0.01$, $^{***}P < 0.001$, $^{****}P < 0.0001$.

Supplementary Figure 11

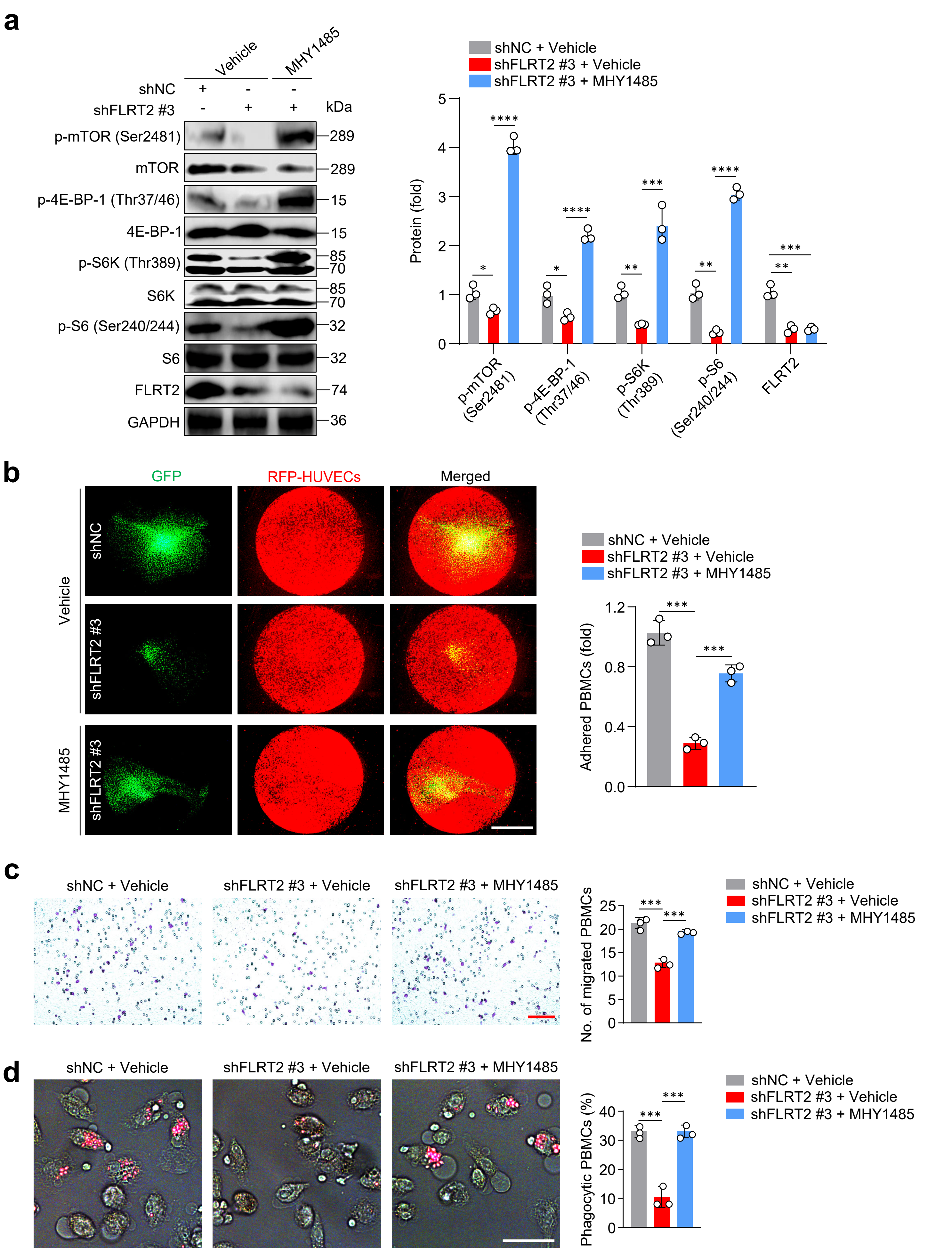

Supplementary Figure 11. MHY1485, an mTOR agonist, improved impaired functions of M-CSF-primed PBMC-derived macrophages with FLRT2 loss. a–d PBMCs were transfected with shNC or shFLRT2 for 48 h, and then incubated with 2 μM MHY1485 for additional 6 h, followed by further analyses. **a** Immunoblot analysis showing p-mTOR (Ser2481), mTOR, p-4E-BP-1 (Thr37/46), 4E-BP-1, p-S6K (Thr389), S6K, p-S6 (Ser240/244), S6, and FLRT2 protein levels in PBMCs indicated above (n = 3). **b** PBMCs were co-cultured with HUVECs expressing RFP for 6 h, followed by fluorescent microscopy (n = 3 mice per group). Scale bar, 5 mm. **c** Transwell cell migration assays were performed, and numbers of migrated cells were counted (n = 3). Scale bar, 100 μm. **d** Phagocytosis assays were performed by culturing the PBMCs indicated above in Texas red-conjugated zymosan particles for 2 h at 37°C (n = 3). Cells were viewed for internalization of the particles by fluorescence microscopy. Scale bar, 50 μm. Data are means ± SD. *P* values were determined using unpaired, two-tailed Student's *t*-tests. $^{*}P < 0.05$, $^{**}p < 0.01$, $^{***}P < 0.001$, $^{****}P < 0.0001$.
